# Current and future immunotherapy for breast cancer

**DOI:** 10.1186/s13045-024-01649-z

**Published:** 2024-12-25

**Authors:** Natalie K. Heater, Surbhi Warrior, Janice Lu

**Affiliations:** 1 Department of Medicine, McGaw Medical Center of Northwestern University, Chicago, IL 60611 USA; 2 Robert H. Lurie Comprehensive Cancer Center of Northwestern University, 676 N St. Clair, Suite 850, Chicago, IL 60611 USA

**Keywords:** Immunotherapy, Immune checkpoint inhibitor, Antibody drug conjugate, Bispecific antibody, Cellular therapy, Breast cancer

## Abstract

Substantial therapeutic advancement has been made in the field of immunotherapy in breast cancer. The immune checkpoint inhibitor pembrolizumab in combination with chemotherapy received FDA approval for both PD-L1 positive metastatic and early-stage triple-negative breast cancer, while ongoing clinical trials seek to expand the current treatment landscape for immune checkpoint inhibitors in hormone receptor positive and HER2 positive breast cancer. Antibody drug conjugates are FDA approved for triple negative and HER2+ disease, and are being studied in combination with immune checkpoint inhibitors. Vaccines and bispecific antibodies are areas of active research. Studies of cellular therapies such as tumor infiltrating lymphocytes, chimeric antigen receptor-T cells and T cell receptor engineered cells are promising and ongoing. This review provides an update of recent major clinical trials of immunotherapy in breast cancer and discusses future directions in the treatment of breast cancer.

## Background

One function of the immune system is to eliminate nascent malignant cells. A hallmark of malignancy is the ability of cancer cells to either actively suppress or escape detection by the immune system. Cancer cells alter their tumor immune microenvironment (TIME), creating an immunosuppressive environment through various mechanisms such as signaling changes, restriction of antigen recognition, and induction of T cell exhaustion [1]. Immune checkpoint proteins, such as programmed-death receptor (PD-1) on T cells, B cells and antigen-presenting cells, PD ligand (PD-L1) on tumor cells, or cytotoxic T-lymphocyte associated protein 4 (CTLA4) are essential components in the host immune response to tumor cells [2]. Mutations in these proteins essentially “turn off” the immune system response to malignant cells. The function of immunotherapy delivered through immune checkpoint inhibitors (ICIs), then, is to restore the normal immune response, allowing the host immune system to destroy malignant cells. ICIs include monoclonal antibodies targeting PD-L1 (atezolizumab, avelumab, and durvalumab), PD-1 (pembrolizumab and nivolumab) or CTLA-4 (Ipilumumab) [3]. Lymphocyte Activation Gene 3 (LAG-3), a recently-discovered immune checkpoint which is found on T cells that have been activated but exhausted, has also been targeted by immunotherapy [4]. In addition, cellular therapies offer a distinct mechanism of immunotherapy with the goal of inciting a durable immunologic response to cancer antigens.

The role of immunotherapy in breast cancer has expanded in the past two decades. Breast cancer was classically considered to be immunologically “cold,” unresponsive to immune manipulations. Ongoing research hopes to identify patients who might have enhanced benefit to immunotherapy based on their individual TIME.

Antibody–drug conjugates (ADCs) are another recent advancement in breast cancer. ADCs utilize monoclonal antibodies conjugated to cytotoxic agents (referred to as the “payload”) at higher concentrations into antigen-expressing tumor cells [5]. This has the benefit of targeting traditional chemotherapies to malignant cells, reducing the risk of off-target systemic toxicities. Some ADCs exhibit the bystander effect, in which cells adjacent to the antigen-expressing tumor cell are also killed by the payload [6]. Common ADC targets in breast cancer include TROP2, HER2, and HER3. While ADCs are not defined as immunotherapy, this new class of agents has been safely combined with ICIs in clinical trials [7]. Research into combinations of ADCs with ICIs is ongoing.

Cancer vaccines are being investigated for both prevention and treatment of breast cancer. A principal target of cancer vaccines are tumor-associated antigens (TAAs), which are proteins that are expressed in both normal tissue and malignant cells [8]. Examples of TAAs in breast cancer include HER2, Muc-1, and CEA [8, 9]. Cancer vaccines stimulate CD4+ T helper cells and CD8+ cytotoxic T lymphocytes to engage with TAAs to induce a specific adaptive immunologic response and to initiate immunologic memory to protect against further exposure to the antigens. A benefit of cancer vaccines is the low adverse effects associated with treatment, though studies have found minimal clinical activity. Possible mechanisms of resistance include downregulation of activated tumor antigens and antigen-presenting cells by the TIME, as well as the destruction of activated T-cells [10].

**Fig. 1:**
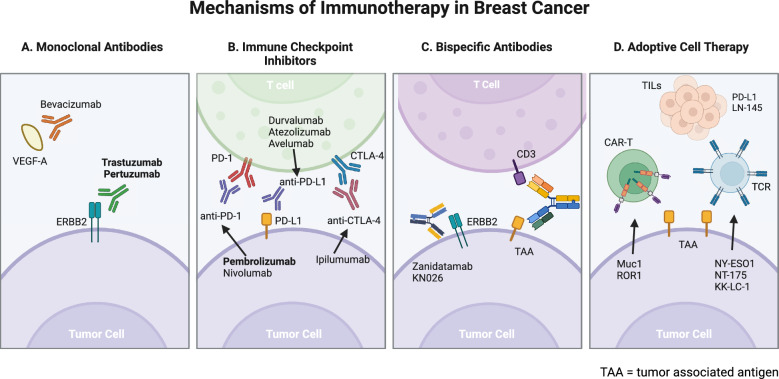
Mechanisms of Current and Upcoming Immunotherapy in Breast Cancer. Therapies in bold are FDA approved. Created in BioRender. https://BioRender.com/t90y911

The role of immunotherapy in breast cancer has expanded in the past two decades. Breast cancer was classically considered to be immunologically “cold,” unresponsive to immune manipulations. Ongoing research hopes to identify patients who might have enhanced benefit to immunotherapy based on their individual TIME (Fig. 1).

Across multiple cancer types, responsiveness to ICIs has been shown to correlate with higher PD-L1 expression on tumor cells, as determined by immunohistochemistry (IHC) [11]. Two of the most common methods of reporting levels of PD-L1 expression are the combined positive score (CPS) and immune-cell score (IC). CPS measures the proportion of PD-L1 positive cells in both tumor and immune cells out of all cells in a tumor sample, as assessed by Dako assay 22C3 and reported as a maximum score out of 100 [12]. IC is defined by the proportion of tumor area occupied by PD-L1-staining immune cells regardless of staining intensity and is assessed by Ventana assay SP142 [12, 13]. Studies of pembrolizumab typically utilize CPS, while studies of atezolizumab most commonly use IC. Concordance between assays is suboptimal, with IC typically reporting a more conservative estimate of PD-L1 positivity [14]. Standard cutoffs for PD-L1 positivity (PD-L1+) include CPS ≥ 1 and IC ≥ 1% [12–14]. PD-L1 positivity is used clinically to determine benefit of pembrolizumab in mTNBC [15].

Another biomarker that may predict response to ICIs in breast cancer is the presence of tumor infiltrating lymphocytes (TILs), such as CD4+ T cells, CD8+ T cells, NK cells, and Th1, which cause an anti-tumor effect through IFN-γ signaling [16, 17]. Recent clinical trials suggest an enhanced benefit for immunotherapy in patients with high levels of TILS, but this is not yet a standard biomarker in clinical practice. Biomarkers for immunotherapy response primarily utilized outside of breast cancer include high tumor mutational burden (TMB), defined as the number of non-inherited mutations per million base pairs, as well as deficiencies in mismatch repair genes (dMMR) which causes increase mutations in microsatellites, termed microsatellite instability-high (MSI-H), though these biomarkers are typically employed in gastrointestinal cancers [18–20]. Negative biomarkers such as TAMs, MDSc, CD4+ TRegs, B2M, HLA-A deletions and JAK1/2 mutations may decrease a cancer’s response to immunotherapy but are not used in clinical practice [21–23].

Immune-related adverse events (irAEs) are thought to be caused by disruptions in self-tolerance, or a non-specific autoinflammatory reaction [24]. The most common irAEs are cutaneous (maculopapular rash, pruritus, or lichenoid dermatitis), followed by gastrointestinal (diarrhea and colitis), then endocrine (hypothyroidism, hypophysitis or adrenal insufficiency) [25]. Rare but fatal irAEs include pneumonitis, myocarditis, hepatitis, and enteritis [25]. Management of irAE typically includes high-dose corticosteroids, with immunosuppressive therapies in severe cases [26]. Permanent discontinuation of immunotherapy is required for all grade 4 irAEs with the exception of endocrinopathies that can be controlled with hormone replacement [26].

Resistance to immunotherapy is a complex process that is possible due to both intrinsic and extrinsic tumor effects. This can occur due to multifactorial changes in the TIME. Causes of resistance include activation of immune checkpoint pathways that suppress any response from cancer cells as well adaptation within the immune recognition cascade to prevent a response by the immune system [27]. These are often referred to as “cold tumors” as they are difficult to be penetrated by immune cells. There is also the possibility of acquired resistance which occurs after an initial response to immunotherapy, and development of genetic mutations leading to resistance. There are also extrinsic factors such as patient characteristics and lifestyle that can lead to resistance as well. Strategies to overcome resistance to immunotherapy is an ongoing investigation. While there is significant improvement in responses with combination dual immunotherapy, addition of targeted therapy, or PD-L1 down regulators/ inhibitors that are being used in other cancers, there needs to be further research in breast cancer to identify ways to overcome immunotherapy resistance [28].

## Triple negative breast cancer (TNBC)

TNBC is an aggressive subtype of breast cancer, accounting for 12–15% of all breast cancer in the United States [29]. In comparison to hormone receptor positive (HR+) breast cancer, TNBC is known to have higher levels of PD-L1 expression, with a median PD-L1 CPS of 7.5 and 50% expressing ≥ CPS 10 [30]. In addition, TNBC has higher levels of TILs and TMB, which are associated with an enhanced response to immunotherapy [18, 22, 23]. Over the past decade, clinical trials in TNBC have incorporated ICIs in combination with traditional cytotoxic chemotherapy with favorable results. In the metastatic setting, response to ICIs is dependent on the degree of PD-L1 expression, whereas in the neoadjuvant/adjuvant setting benefit to ICIs is seen regardless of PD-L1 positivity. This is thought to be explained, at least in part, by decreasing PD-L1 expression and TILs over the course of disease progression and with higher burden of disease [31].

### Advanced and metastatic TNBC

The first studies of immunotherapy in breast cancer were conducted as monotherapy in treatment-refractory, PD-L1+ advanced/metastatic TNBC (a/mTNBC). The 2016 KEYNOTE-012 phase 1b trial showed an encouraging overall response rate (ORR) of 18.5% for patients with PD-L1 ≥ 1% mTNBC treated with single-line pembrolizumab [32]. This was followed by the phase II KEYNOTE-086 of single-agent pembrolizumab in mTNBC, which found an ORR of 21% when used in the first line for PD-L1 positive disease, and a more modest ORR of 5.7% after the first line in any level of PD-L1 expression [33, 34]. These results suggested a benefit from incorporating ICIs in PD-L1+ disease, with an enhanced benefit earlier in the treatment course. The phase III KEYNOTE-119 then compared single-agent pembrolizumab to chemotherapy of choice (capecitabine, gemcitabine, eribuilin, or vinorelbine) in 622 patients in the second- or third-line setting. While no difference in progression free survival (PFS) or overall survival (OS) was seen, a graded ORR to pembrolizumab was seen with higher PD-L1 CPS, with the most benefit seen in patients with CPS ≥ 10 [35, 36]. In a phase II trial across all solid malignancies with MSI-H or MMR disease including thirteen patients with breast cancer, single-agent pembrolizumab had a median ORR of 30.8% and PFS of 3.5 months [20].

Pembrolizumab in combination with chemotherapy is now the standard first-line therapy for PD-L1+ a/mTNBC based on the KEYNOTE-355 trial. This phase III study of 847 patients compared first-line standard chemotherapy with pembrolizumab or placebo. Patients with PD-L1 CPS ≥ 10 receiving pembrolizumab exhibited a statistically significant improvement in PFS (9.7 months vs. 5.6 months, HR (hazard ratio) 0.65) and OS (23.0 months vs. 16.1 months, HR 0.73) [15, 37], with a trend towards significance for patients with CPS ≥ 1. The PFS benefit was more pronounced in patients receiving paclitaxel or nab-paclitaxel rather than gemcitabine/carboplatin. Patients with a disease-free interval (DFI) of more than 6 months after curative treatment for early-stage TNBC were included. In an exploratory analysis, patients with early recurrence (6–12 months after curative-intent treatment) had less benefit from immunotherapy in combination with chemotherapy (HR 1.44) [15]. The FDA approved pembrolizumab for first-line advanced/metastatic TNBC in 2020 and this regimen is now the standard of care in the first line for patients with PD-L1+ a/mTNBC (Table 1).

**Table 1:** Current and upcoming clinical trials of immune checkpoint inhibitors in advanced/metastatic triple negative breast cancer

Trial Name:	Primary Author	Year	Study Design	Line of Therapy	Setting	Biomarker	# Patients	Drug regimen	Results
*Pembrolizumab*									
NCT01848834 KEYNOTE-012	Nanda	2016	Phase Ib	Heavily Pretreated	Metastatic	PD-L1 ≥ 1%	32	Pembrolizumab	ORR 18.5% (95% CI 6.3–38.1) DCR 25.9% (11.1–46.3)
NCT02447003 KEYNOTE-086	Adams	2018	Phase II	> First line	Metastatic	Any	170	Pembrolizumab	Overall ORR 5.3% (95% CI 2.7–9.9) PD-L1 + ORR 5.7% (2.4–12.2) Overall DCR 7.6% (4.4–12.7) PD-L1 + DCR 9.5% (5.1–16.8)
NCT02447003 KEYNOTE-086	Adams	2018	Phase II	First line	Metastatic	PD-L1 ≥ 1%	84	Pembrolizumab	ORR 21% (95% CI 13.9–31.4) DCR 23.8% (15.9–34.0) PFS 2.1 mo (2.0–2.2) OS 18.0 mo (12.9–23.0)
NCT02628067 KEYNOTE-158 Cohort K	Maio	2022	Phase II	> First line	Advanced/metastatic solid tumors, including breast	MSI-H or dMMR	13	Pembrolizuamb	ORR 30.8% (95% CI 25.8–36.2) PFS 3.5 mo (2.3–4.2) OS 20.1 (14.1–27.1)
NCT02971761	Yuan	2020	Phase II	Any	Metastatic	Androgen Receptor > 10%	18	Enobosarm + Pembrolizumab	RR 13% CBR 25% PFS 2.6 mo (95% CI 1.9–3.1) OS 25.5 mo (10.4-NE)
NCT02513472 ENHANCE 1/KEYNOTE-150	Tolaney	2021	Phase Ib/II	First to third line	Metastatic	Any	167	Eribulin + Pembrolizumab	Overall ORR 23.4% (95% CI 17.2–30.5) Overall PFS 4.1 mo (3.5–4.2) Overall OS 16.1 mo (13.3–18.5) First Line ORR 25.8% (15.8–38) First Line PFS 4.2 mo (3.5–5.5) First Line OS 17.4 mo (13.2–21.0) Second or Third Line ORR 21.8% (14.2–31.1) Second or Third Line PFS 4.1 mo (3.5–4.2) Second or Third Line OS 15.5 mo (12.5–18.7)
NCT02657889 KEYNOTE-162/TOPACIO	Vinayak	2019	Phase II	> First line	Advanced/metastatic	Any	55	Niraparib + Pembrolizumab	ORR 18% (90% CI 10–29); DCR 42% (31–54) BRCAmut ORR 47% (24–70), DCR 80% (56–94) BRCAwt ORR 11% (3–26), DCR 33% (19–51) PD-L1 CPS ≥ 1: ORR 32% (18–49), DCR 50% (33–67)
NCT03106415	Chumsri	2023	Phase I/II	≤ 3 Lines, no prior PD-1 or PD-L1 therapies	Advanced/metastatic	Any	22	Binimetinib + Pembrolizumab	Safety: 3 patients with DLT ORR 29.4 (95% CI 10.3–55.9) with one CR and 4 PR CBR 35.3% (14.2–61.7)
NCT02819518 KEYNOTE-355	Cortes	2020	Phase III	First line	Metastatic	Any	847	Arm A: Nab-Paclitaxel/Paclitaxel/Gemcitabine-Carboplatin + Pembrolizumab Arm B: Nab-Paclitaxel/Paclitaxel/Gemcitabine-Carboplatin + Placebo	**PFS CPS ≥ 10 9.7 vs. 5.6 mo, HR 0.66 (95% CI 0.50–0.88)** PFS CPS ≥ 1 7.6 vs. 5.6 mo, HR 0.75 (0.62–0.91) PFS ITT 7.5 vs. 5.6 mo, HR 0.82 (0.70–0.98) **OS CPS ≥ 10 23.0 vs. 16.1 mo, HR 0.73 (0.55–0.95)** OS CPS ≥ 1 17.6 vs. 16.0 mo, HR 0.86 (0.72–1.04) OS ITT 17.2 vs. 15.5 mo, HR 0.89 (0.76–1.05) irAE 26.5% vs 6.4%
NCT03044730	Shah	2020	Phase II	Any	Metastatic	Any	16	Capecitabine + Pembrolizumab	ORR 13% CBR 15% PFS 4.0 mo (95% CI 1.9–12.7)
NCT02555657 KEYNOTE-119	Winer	2021	Phase III	> First line	Metastatic	Any	622	Arm A: Pembrolizaumab Arm B: Physician's choice of Capecitabine, Eribuin, Gemcitabine or Vinorelbine	ITT OS 9.9 vs. 10.8 mo, HR 0.97 (95% CI 0.82–1.15) CPS ≥ 10 OS 12.7 vs. 11.6 mo, HR 0.78 (0.57–1.06) CPS ≥ 1 OS 10.7 vs. 10.2 mo, HR 0.86 (0.69–1.06) ITT PFS 2.1 vs. 3.3 mo, HR 1.60 (1.33–1.92) CPS > 10 PFS 2.1 vs. 4.3 mo, HR 1.14 (0.82–1.59) CPS ≥ 1 PFS 2.1 vs. 3.1 mo, HR 1.35 (1.08–1.68)
NCT03797326 LEAP-005	Chung	2020	Phase Ib	> FIrst line	Advanced/metastatic	Any	31	Lenvatinib + Pembrolizumab	ORR 29% (95% CI 14–47) DCR 58% (38–76) 55% of patients had grade 3–5 TRAE with one death
NCT03012230	Kassi	2023	Phase I	Heavily pretreated	Metastatic	Any	12	Pembrolizumab + Ruxolitinib	5 patients with grade 3 or higher AE; MTD not established 2 patients with stable disease lasting 6 mo
NCT02411656	Iwase	2023	Phase II	First Line maintenance	mTNBC or Inflammatory	Any	43	Pembrolizumab	4-mo DCR 58.1% (95% CI 43.4–72.9) PFS 4.8 mo (3.0–7.1)
NCT04191135 KEYLYNK-009	Rugo	2020	Phase II	First Line maintenance	Metastatic	Any	271	Arm A: Olaparib + Pembrolizumab Arm B: Gemcitabine + Carboplatin + Pembrolizumab	PFS 5.5 vs. 5.6 mo, HR 0.98 (95% CI 0.72–1.33) OS 25.1 vs. 23.4 mo, HR 0.95 (0.64–1.40) PD-L1 ≥ 10% PFS 5.7 vs. 5.7 mo, HR 0.92 (0.59–1.43) PD-L1 ≥ 10% OS NE vs. NE BRCA + PFS 12.4 vs. 8.4 mo, HR 0.7 (0.33–1.48) BRCA + OS NE vs. 23.4 mo (17.3-NE)
NCT02734290	Page	2023	Phase Ib	First or second line	Metastatic	Any	29	Arm A: Paclitaxel + Pembrolizumab Arm B: Capecitbaine + Pembrolizumab	ORR 29% (95% CI 10–61) vs 43% (18–71) PFS 83 vs 155 days
NCT02981303 IMPRIME 1	O'Day	2020	Phase II	> First line	Metastatic	Any	44	Odetiglucan + Pembrolizumab	ORR 15.9% (95% CI 4.9–29.4) DCR 54.5% (40.1–68.3) 12 mo OS 57.6% (42.4–72.8) mOS 16.4 mo (11.1–23.9)
*Atezolizumab*									
NCT01633970	Adams	2019	Phase Ib	Any	Metastatic	Any	33	Atezolizumab + Nab-Paclitaxel	ORR 39.4% (95% CI 22.9–57.9) DCR 51.5% (33.5–69.2) PFS 5.5 mo (5.1–7.7) OS 14.7 mo (10.1-NE)
NCT02425891 IMpassion130	Schmid	2018	Phase III	First line	Metastatic	PD-L1 positive	902	Arm A: Atezolizumab + Nab-Paclitaxel Arm B: Placebo + Nab-Paclitaxel	**PFS 7.2 vs. 5.5 mo, HR 0.80 (95% CI 0.69–0.92)** **PD-L1 ≥ 1% PFS 7.5 vs. 5.0 mo, HR 0.62 (0.49–0.78)** OS 21.3 vs. 17.6 mo, HR 0.84 (0.69–1.02) PD-L1 ≥ 1% OS 25.0 vs. 15.5 mo, HR 0.62 (0.45–0.86) irAE 57.3% vs 41.8% Grade 3 + irAEs 7.5% vs 4.3%
NCT01375842	Emens	2019	Phase I	Any	Metastatic	Any	116	Atezolizumab	First-Line ORR 24% (95% CI 8.2–47.2) First-Line OS 17.6 mo (10.2-NE) ≥ Second-Line ORR 6% (2.4–13.4) ≥ Second-Line OS 7.3 mo (6.1–10.8)
NCT02322814 COLET	Brufsky	2021	Phase II	First line	Advanced/metastatic	Any	153	Cohort I: Arm A: Paclitaxel + Cobimetinib Arm B: Paclitaxel + Placebo Cohort II: Atezolizumab + Cobimetinib + Paclitaxel Cohort III: Atezolizumab + Cobimetinib + Nab-Paclitaxel	Cohort I PFS 5.5 vs 3.8 mo, HR 0.73 (95% CI 0.43–1.24) Cohort I Arm A ORR 38.3% (24.40–52.20) Cohort I Arm B ORR 20.9% (8.77–33.09) Cohort II ORR 34.4% (18.57–53.19) Cohort III ORR 29.0% (14.22–48.04)
NCT02849496	Fanucci	2023	Phase II	Any	Advanced / metastatic HER2 negative	BRCA1 or BRCA2 mutant	78	Arm A: Atezolizumab + Olaparib Arm B: Placebo + Olaparib	Overall PFS 7.67 (95% CI 5.6–10) vs. 7.0 mo (5.5–11.5) (*p* = 0.92) OS 26.5 (19.2-NE) vs 22.4 mo (16.6–31.3) (*p* = 0.3)
NCT03125902 IMpassion131	Miles	2021	Phase III	First line	Metastatic	Any	651	Arm A: Atezolizumab + Paclitaxel Arm B: Placebo + Paclitaxel	ITT PFS 5.7 vs. 5.6 mo, HR 0.86 (95% CI 0.70–1.05) ITT OS 19.2 vs. 22.8 mo, HR 1.12 (0.88–1.43) PD-L1 ≥ 1% PFS 6.0 vs. 5.7 mo, HR 0.82 (0.60–1.12) PD-L1 ≥ 1% OS 22.1 vs. 28.3 mo, HR 1.11 (0.76–1.64) irAE 62% vs 53%
NCT03829501	Patel	2021	Phase I/II	Heavily pretreated	Metastatic solid malignancy, including TNBC	Any	69	Atezolizumab + KY1044	One CR, one PR
NCT03371017 IMpassion132	Dent	2024	Phase III	First relapse	Advanced, Early-Relapsing TNBC	Any	354	Arm A: Atezolizumab with Gemcitabine/Carboplatin or Capecitabine Arm B: Placebo with Gemcitabine/Carboplatin or Capecitabine	PD-L1 OS 12.1 vs. 11.2 mo, HR 0.93 (95% CI 0.73–1.20) OS 10.4 vs. 9.8 mo, HR 0.94 (0.76–1.18)*
NCT03101280 COUPLET	Kristeleit	2024	Phase Ib/II	> First line	Metastatic	BRCA1 or BRCA2 mutant or BRCAwt/LOH high	5	Atezolizumab + Rucaparib	Safety: 2 of 5 patients experienced grade 3 or 4 AE ORR 40% (95% CI 5–85%), two PR
NCT02708680 ENCORE-602	O'Shaughnessy	2020	Phase II	Third line	Metastatic (TNBC or HR + /HER2 +)	Any	81	Arm A: Atezolizumab + Entinostat Arm B: Atezolizumab + Placebo	PFS 1.68 vs 1.51 mo, HR 0.89 (95% CI 0.53–1.48)
NCT04408118 ATRACT1B	Gion	2023	Phase II	First line	Advanced/metastatic	Any	100	Atezolizumab + Bevacizumab + Paclitaxel	PFS 11.0 mo (95% CI 9.0–13.2) ORR 63% CBR 79%
NCT04177108 IPATunity170 + NCT03337724 IPATunity130 + NCT03800836 CO40151	Schmid	2024	Phase Ib-III	First line	Advanced/metastatic	Any	317	Atezolizumab + Ipatasertib + Paclitaxel/Nab-Paclitaxel +	ORR 44%–63% mPFS 5.4–7.4 mo mDOR 5.6–11.1 mo mOS 15.7–28.3 mo
*Avelumab*									
NCT01772004 JAVELIN	Dirix	2018	Phase Ib	Heavily Pretreated	Metastatic	Any	58	Avelumab	Overall ORR 5.2% PD-L1 ≥ 1% ORR 22.2%
*Camrelizumab*									
NCT03805399 FUTURE Arm C	Liu	2023	Phase II	Heavily pretreated	Metastatic	Immunomodulatory	46	Camrelizumab + Nab-Paclitaxel	ORR 43.5% (95% CI 28.9–58.9) mPFS 4.6 mo (3.4–5.9) mOS 16.1 mo (11.7–20.5) mDOR 8.6 mo (1.2–19.7)
NCT04129996 FUTURE-C-PLUS	Chen	2022	Phase II	First line	Advanced/metastatic	Immunomodulatory	48	Camrelizumab + Famitinib + Nab-Paclitaxel	ORR 81.3% (95% CI 70.2–92.3) mPFS 13.6 mo (8.4–18.8) mDOR 14.9 mo (NC–NC)
NCT04395989 FUTURE-SUPER	Fan	2024	Phase II	First line	Advanced/metastatic	Immunomodulatory	139	Arm A: Camrelizumab + Famitinib + Nab-Paclitaxel Arm B: Nab-Paclitaxel	PFS 15.1 vs. 6.5 mo, HR 0.46 (95% CI 0.25–0.85)
*Durvalumab*									
NCT02734004 MEDIOLA	Domchek	2020	Phase I/II	> Third line	Metastatic HER2- (TNBC or HR +)	BRCA1 or BRCA2 mutant HER2-negative	34	Dulvalumab + Olaparib	ORR 63.3% (95% CI: 48.9–80.1) DCR at 12 weeks 80% (90% CI: 64.3–90.0) DCR at 28 weeks 50% (90% CI: 33.9–66.1)
NCT02299999 SAFIR02-BREAST IMMUNO	Bachelot	2021	Phase II	First line maintenance	Metastatic	Any	82	Arm A: Durvalumab Arm B: Chemotherapy	**mOS 14.0 vs 21.1 mo, HR 0.54 (95% CI 0.30–0.97)** PD-L1 ≥ 1% mOS 27.3 vs. 12.1 mo, HR 0.37 (0.12–1.13)
NCT03167619 DORA	Tan	2024	Phase II	First Line maintenance	Advanced/metastatic	Any	45	Arm A: Olaparib Arm B: Durvalumab + Olaparib	PFS 4.0 (95% CI 2.6–6.1) vs 6.1 mo (3.7–10.1) CBR 44% (23–66) vs 36% (17–59)
*Nivolumab*									
NCT02499367 TONIC	Voorwerk	2019	Phase I/II	Heavily pretreated	Metastatic	Any	67	Arm A: waiting period then Nivolumab Arm B: irradiation then Nivolumab Arm C: Cyclophosphamide then Nivolumab Arm D: Cisplatin then Nivolumab Arm E: Doxorubicin then Nivolumab	Overall ORR 20% Arm A ORR: 17% Arm B ORR: 8% Arm C ORR: 8% Arm D ORR: 23% Arm E ORR: 35%
WJOG9917B NEWBEAT	Ozaki	2022	Phase II	First line	Metastatic	Any	17	Bevacizumab + Nivolumab + Paclitaxel	ORR 59% PFS 7.8 mo
NCT02637531 MARIO-1	Hong	2023	Phase I	Previously treated	Metastatic	Any	29	Part F: Eganelisib + Nivolumab	ORR 7%; one CR, one PR DCR 30%
*Toripalimab*									
NCT03777579 TORCHLIGHT	Jiang	2024	Phase III	First line	Advanced/metastatic	Any	531	Arm A: Nab-Paclitaxel + Toripalimab Arm B: Nab-Paclitaxel + Placebo	**PD-L1 CPS ≥ 1 PFS 8.4 vs. 5.6 mo, HR 0.65 (95% CI 0.470–0.906)** ITT PFS 8.4 vs. 6.9 mo, HR 0.77 (0.602–0.994) PD-L1 CPS ≥ 1 OS 32.8 vs. 19.5 mo, HR 0.62 (0.414–0.914) ITT OS 33.1 vs. 23.5 mo, HR 0.69 (0.513–0.932)
*LAG-3 modulation*									
NCT00349934	Brignone	2010	Phase I/II	First line	Metastatic	Any	30	Eftilagimod + Paclitaxel	ORR 50%
NCT02460224	Lin	2024	Phase II	> First line	Advanced/Metastatic	Any	56	Arm A: Leramilimab + Spartzlizumab (q3W) Arm B: Leramilimab + Spartzlizumab (q4W)	ORR 9.5%; response only in PD-L1 positive
*Dual Immunotherap*									
	Santa Maria	2018	Pilot study	> First line	Metastatic	Any	7	Durvalumab + Tremelimumab	ORR 43% Hepatotoxicity major AE
NCT02834013 DART/SWOG S1609 Cohort 36	Adams	2022	Phase II	Any	Metaplastic	Any	17	Ipilumumab + Nivolumab	ORR 18% 3 responders all had ongoing response at 28 + mo. All responders had adrenal insufficiency
NCT03789110 NUMBUS	Barrosa-Sousa	2020	Phase II	Any	Metastatic HER2-	TMB-High	31	Ipilumumab + Nivolumab	ORR 13.3% PFS 1.4 mo (95% CI 1.3–9.5) OS 8.8 mo (95% CI 4.2–NE) Exploratory: TMB ≥ 14 mut/Mb ORR 60% vs 9–14 ORR 4% (*p* = 0.01) No grade 4/5 toxicities
NCT03650894	Page	2023	Phase II	First or Second line	Metastatic HER2-	Any	30	Ipilumumab + Nivolumab + Bicalutamide	HR+ /Androgen Receptor Negative CBR 8% HR-/AR+ CBR 33%
NCT02453620	Roussos Torres	2024	Phase Ib	> First line	Metastatic	Any	12	Entinostat + Ipilumumab + Nivolumab	No DLT ORR 40% (95% CI 12.2–73.8) CBR 60% (95% CI 26.2–87.8)
*Selected Upcoming Clinical Trials*									
NCT04739670 BELLA			Phase II	First Relapse	Advanced, Early-Relapsing	PD-L1+	31	Atezolizumab + Bevacizumab + Carboplatin + Gemcitabine	PFS
NCT04148911 EL1SSAAR			Phase IIIb	First line	Advanced/metastatic	PD-L1+	184	Atezolizumab + Nab-Paclitaxel	Safety
NCT03961698 MARIO-3			Phase II	First line	Advanced/metastatic	Any	91	Atezolizumab + Bevacizumab + Eganelisib + Nab-paclitaxel	CRR
NCT03915678 ADAGIR			Phase II	Heavily pretreated	Metastatic solid tumors, including TNBC	Any	247	Atezolizumab + BDB001 + stereotactic radiation	DCR

Results in bold are statistically significant

Atezolizumab as monotherapy in the first-line setting for a/mTNBC was found to have an ORR of 24% [38] and an ORR of 39.4% when combined with nab-paclitaxel after 0–2 lines of prior therapy [39]. The phase III IMpassion130 study randomized 902 patients with advanced PD-L1+ TNBC at least one year from curative-intent therapy or de novo metastatic disease to nab-paclitaxel with atezolizumab or placebo. Patients who received nab-paclitaxel with atezolizumab had a significant PFS benefit compared to patients who received nab-paclitaxel with placebo (7.2 months vs. 5.5 months, HR 0.80) in the intention-to-treat (ITT) analysis and in the PD-L1 > 1% subgroup (7.5 months vs. 5.0 months, HR 0.62) [11]. While these results did not translate into a significant OS benefit in the ITT analysis, a median 9.5-month OS benefit was seen in the PD-L1 > 1% subgroup (HR 0.62, significance not evaluated due to hierarchical testing plan) [11].

Atezolizumab in combination with other chemotherapy backbones has found less success. The IMpassion131 phase III trial investigated paclitaxel + atezolizumab or placebo in 651 patients, with no difference in PFS or OS in either the ITT to PD-L1 subgroup [40], though it is noted that the control group experienced an unprecedented OS of 22.8 months, which may have impacted the results. Overall, the reasons for discrepant results between IMpassion130 and IMpassion131 are unclear. Possible explanations include different formulations between paclitaxel and nab-paclitaxel, pretreatment with steroids in the IMpassion131 study, or differences in the tumor microenvironment in each study population [40, 41]. Though the FDA initially approved atezolizumab based on the results of IMpassion 130, the approval was removed after the IMpassion 131 results were reported.

The possible benefit of atezolizumab in early-relapsing TNBC, a high-risk population defined as relapse less than twelve months after last chemotherapy or surgery for early-stage disease, was evaluated in the phase III IMpassion132 study. In this study, 354 patients without prior immunotherapy were randomized to chemotherapy of physician’s choice plus either atezolizumab or placebo. Initially, patients with any PD-L1 status were included, which was later restricted to patients with PD-L1 > 1%. Two thirds of patients had a DFI of < 6 months. No difference in median disease-free interval or OS was seen [42]. These results, in combination with the exploratory analysis of relapse < 12 months in KEYNOTE-355, suggest that some patients with quickly relapsing TNBC may have an intrinsic resistance to immunotherapy. However, as discussed in greater depth below, the standard of care for first-line therapy in early-stage TNBC now includes immunotherapy, and as such few patients will reach the early-relapsed setting without prior immunotherapy.

The Atract1B phase II trial challenged the view that ICIs only have benefit in PD-L1 positive disease. This trial investigated paclitaxel, atezolizumab and bevacizumab (a VEGF-inhibitor) in the first line for advanced TNBC, with 97% of patients having PD-L1 negative disease. Median PFS was 11.0 months, with an ORR of 63%, including thirteen complete responses and 50 partial responses [43]. Bevacizumab in combination with nivolumab and paclitaxel was investigated in the first line of patients with metastatic HR + /HER- or TNBC in the NEWBEAT phase II trial, with an ORR of 70% (59% in TNBC, 74% in HR + /HER-) [44].

Clinical trials of dual ICI therapy in mTNBC have shown some clinical benefit but also raise concerns of higher rates of toxicity. Durvalumab in combination with tremelimumab, an anti-CTLA-4 antibody, was found to have an ORR of 43% in TNBC in a pilot study of 7 patients [45]. The DART/SWOG S1609 phase II trial of ipilumumab with nivolumab found an ORR of 18%, though all patients who had an initial response continued to respond nearly 3 years later. All responders developed adrenal insufficiency [46].

The use of Poly (ADP-ribose) polymerase inhibitors (PARPi) in combination with ICIs for patients with BRCA-mutated disease has shown promise in phase II trials. Olaparib with durvalumab had a 63.3% ORR in patients with heavily pretreated BRCA-mutant HER2-negative metastatic breast cancer (mBC) with an 80% 3-month disease control rate [47]. However, a trial of olaparib with or without atezolizumab in BRCA-mutant a/m TNBC found no PFS or OS benefit for combination therapy but did have more adverse effects [48]. The TOPACIO/KEYNOTE-162 trial evaluated patients with a/mTNBC with any PD-L1 status. Patients were treated with niraparib and pembrolizumab, with higher ORR seen in patients with BRCA-mutated disease (47%) compared to BRCA-wild type (11%), with updated PFS and OS not yet reported [49].

Optimizing maintenance regimens for patients with initial response to chemotherapy is an active area of research. The DORA phase II study evaluated the role of maintenance olaparib with or without durvalumab in patients with aTNBC who responded to platinum-based chemotherapy. Patients experienced a median PFS of 4.0 months versus 6.1 months, with benefit seen regardless of BRCA or PD-L1 status [50]. The KEYLYNK-009 phase II trial investigated the efficacy of maintenance pembrolizumab and olaparib compared to pembrolizumab and chemotherapy in patients with recurrent inoperable or mTNBC who responded to induction pembrolizumab and chemotherapy. No difference in PFS after completion of induction therapy (5.5 months vs. 5.6 months) or OS (25.1 months vs. 23.4 months) was seen, though there was a trend toward improved PFS for patients with BRCA-mutated disease [51]. Interestingly, no improvement in patient-reported outcomes was seen for patients who were maintained on a chemotherapy-free regimen in comparison with standard pembrolizumab and chemotherapy [52]. Another study of maintenance immunotherapy with durvalumab in comparison to chemotherapy found a 7.1 month OS benefit with durvalumab in an exploratory analysis of patients with TNBC [53]. Overall, these studies suggest an emerging role for chemotherapy-free maintenance for patients who have an initial response to chemotherapy.

Sacituzumab govitecan (SG), an ADC consisting of an anti-TROP2 antibody linked to a topoisomerase I inhibitor, was compared to physician’s choice of treatment in the second or third line of a/m TNBC in the phase III ASCENT trial, with a significant improvement in PFS (4.8 months vs 1.7 months, HR 0.41) and a 4.9 month absolute OS benefit (11.8 vs 6.9 mo, HR 0.51), leading to early termination for efficacy [54, 55]. SG gained FDA approval for mTNBC after two prior therapies in April 2020 [54]. SG in combination with atezolizumab is being compared to the IMPassion030 regimen of nab-paclitaxel + atezolizumab in the front line for PD-L1+ a/m TNBC in the MORPHEUS-pan BC trial, with preliminary data showing an encouraging ORR of 76.7% versus 66.7% and immature PFS data of 12.2 months versus 5.9 months (HR 0.27) [7].

Datopotamab deruxtecan (Dato-DXd) is another anti-TROP2 antibody linked to a topoisomerase inhibitor that has shown activity in TNBC. This ADC was investigated in the phase I TROPION-Pan Tumour 01 study, which found an ORR of 31.8% in patients with heavily pretreated a/m TNBC [56]. Dato-DXd is being studied in combination with an ICI in the phase Ib/II BEGONIA trial, with arm 7 of this trial investigating Dato-DXD with durvalumab in the first line for a/m TNBC. Early results found an ORR of 79% ORR, with 47% of patients having an ongoing response at 11.7 months and response seen regardless of level of PD-L1 expression [57]. Other ADCs being investigated in mTNBC include enfortumab vedotin, which found an ORR of 195 and PFD of 3.5 months in heavily-pretreated mTNBC in the phase II EV-202 trial [58]. Another anti-TROP2 ADC of note is Sacituzumab tirumotecan, which was found to significantly improved OS versus chemotherapy of physician’s choice in the second line for a/m TNBC [59].

### Early Stage TNBC

Neoadjuvant treatment of early stage TNBC aims to reduce the extent of surgical excision for operable tumors or convert inoperable tumors to operable tumors. The treatment goal is to attain a pathological complete response (pCR), defined as the eradication of invasive cancer from the breast and lymph nodes (ypT0/is, ypN0) at the time of surgery [60]. Patients who achieve pCR experience significantly improved disease-free survival (DFS) and OS outcomes, thus it is a common clinical end point in neoadjuvant trials [61]. Patients failing to achieve pCR are classified according to the degree of residual cancer burden (RCB). The degree of RCB is also prognostic, with higher RCB scores prognostic for worse event-free survival (EFS) [62].

Combining chemotherapy with ICI increases the rate of pCR for patients with TNBC in comparison to standard neoadjuvant chemotherapy regimens. Unlike the metastatic setting in which PD-L1 is predictive of response to ICIs, the development of predictive biomarkers in the neoadjuvant setting is elusive. As such, there is currently no indication for PD-L1 testing outside of clinical trials as the current evidence shows benefit for ICIs for all early TNBC in the neoadjuvant setting, regardless of PD-L1 status.

Pembrolizumab is FDA approved for neoadjuvant therapy of early-stage TNBC. The phase 1b KEYNOTE-173 trial demonstrated safety and preliminary efficacy of pembrolizumab in the first line with neoadjuvant chemotherapy for early-stage, high-risk TNBC, with higher PD-L1 CPS and TILs significantly associated with higher rates of pCR [16, 63]. Data from the phase II I-SPY2 trial established the benefit of pembrolizumab added to standard neoadjuvant chemotherapy. Twenty-nine patients with TNBC > 2.5 cm and any nodal status were included. Patients in the experimental arm were treated with pembrolizumab with weekly paclitaxel followed by dose-dense (dd) doxorubicin and cyclophosphamide (AC), with estimated pCR rates of 60% vs. 22% for patients treated with paclitaxel followed by AC [64]. Another I-SPY2 regimen evaluated paclitaxel with pembrolizumab in an anthracycline-free regimen, but did not reach target pCR rates [65].

The landmark phase III, double-blind KEYNOTE-522 trial evaluated 1174 patients with cT1, N1-2 or cT2-4, N0-2 TNBC with the goal of investigating the efficacy of neoadjuvant and adjuvant pembrolizumab. Patients were randomized 2:1 to receive neoadjuvant pembrolizumab or placebo with paclitaxel and carboplatin (PC) followed by AC or epirubicin and cyclophosphamide (EC) (every-3-week dosing). After surgery, patients in the study group continued pembrolizumab to complete one total year of treatment. The pembrolizumab regimen was associated with a pCR rate of 64.8% vs. 51.2% in the placebo group, representing a treatment difference of 13.6 percent [66]. Moreover, the risk of recurrence was significantly lower in the pembrolizumab group (HR 0.63). More benefit was seen for patients with node-positive disease (treatment difference 20.6% [8.9–31.9] vs. 6.3% [− 5.3–18.2]), with no difference in response based on PD-L1 status. An updated 5-year EFS showed continued benefit for pembrolizumab with EFS rates of 81.2% versus 72.2% [67]. Improved EFS was seen even for patients with RCB-I and RCB-II after neoadjuvant chemotherapy, though patients with RCB-III after neoadjuvant therapy with pembrolizumab had worse 3-year EFS than patients who received placebo (26.2% vs. 34.6%, HR 1.24 [0.69–2.23]) [68]. This decrease in survival was driven by a higher rate of local recurrence. Five-year OS was 86.6% versus 81.7% [69]. Immune-related adverse effects occurred in 33.5% of patients receiving pembrolizumab, most commonly hypothyroidism (15.1%), skin reactions (5.7%) and adrenal insufficiency (2.6%) [70]. Based on this study, the FDA granted approval for neoadjuvant and adjuvant pembrolizumab in 2021. The KEYNOTE-522 regimen is the current standard of care for early stage TNBC.

Given the high rates of pCR and EFS seen with the Keynote-522 regimen of pembrolizumab with PC + AC, the NeoPACT trial evaluated the role of de-escalating anthracyclines in the neoadjuvant setting in an effort to decrease anthracycline toxicities. In this phase II trial, patients receiving carboplatin with docetaxel and pembrolizumab had a pCR rate of 58%. Patients achieving pCR experienced an impressive 3-year EFS of 98%, with 3-year EFS 86% overall [71]. The currently-enrolling phase III SWOG2212 / SCARLET (NCT05929768) trial will compare the KEYNOTE-522 regimen with the NeoPACT regimen in patients with T2-4, N0, M0 or T1-3, N1-2, M0 TNBC with a primary endpoint of EFS, with the goal to establish an optimal chemotherapy backbone.

Neoadjuvant atezolizumab has also shown promise in TNBC. Neoadjuvant atezolizumab demonstrated a pCR benefit when added to an anthracycline-free regimen of carboplatin and paclitaxel in a phase II trial of 67 patients [72]. The phase III IMpassion031 study of 333 patients with cT2-4, N0-3 TNBC found a significant pCR benefit for neoadjuvant atezolizumab with nab-paclitaxel followed by ddAC (pCR 58% vs. 41%). In the PD-L1 cohort, rates of pCR were significantly increased with atezolizumab (68.8% vs. 49.3%) [73]. EFS data is pending. The NeoTRIPaPDL1 / Michelangelo phase III study evaluated carboplatin and nab-paclitaxel with and without atezolizumab followed by surgery and adjuvant AC in 280 patients, without a pCR benefit seen in the atezolizumab group (48.6% vs. 44.4%) [74]. Unlike the KEYNOTE-522 study, the NeoTRIP study included patients with N3 disease, with 88% of all patients having node-positive disease, which may explain lower overall rates of pCR. A multivariate analysis found PD-L1 expression to significantly increase rates of pCR (OR 2.08, 95% CI 1.64–2.65) [74]. EFS data is pending.

Based on the OS benefit seen with maintenance durvalumab in mTNBC [53], the phase II GeparNuevo trial investigated neoadjuvant durvalumab with chemotherapy. Patients with cT2-4d, N0-3 TNBC were randomized to a window period of either durvalumab or placebo, followed by the same treatment combined with nab-paclitaxel, followed by dd EC with durvalumab or placebo. While there was no significant pCR benefit (53% vs. 44%, OR 1.45 [0.80—2.63] [22], durvalumab did show an increased 3-year DFS (85.6% vs. 77.2%, HR 0.48, [0.24–0.97]), supporting the hypothesis of long-term benefits of early ICI without adjuvant ICI [75]. In a subgroup analysis, TMB > 10% and the presence of TILs predicted treatment response, with pCR rates of 82% seen in patients with high TMB and TILs in comparison to pCR rates of 28% in patients with low TMB and TILs [18]. Data from I-SPY2 supports further investigation of neoadjuvant paclitaxel, durvalumab and olaparib followed by AC in early-stage TNBC, as this trial found pCR rates of 47% compared to 27% in the standard therapy arm [76].

Biomarkers to identify patients who are likely to respond to neoadjuvant immunotherapy are needed. Novel biomarkers such as DetermaIO utilize RNA sequencing to produce a score which predicts pCR of early stage TNBC when treated with immunotherapy [77]. A pooled analysis of 343 patients treated in one of 5 immunotherapy arms of the I-SPY2 trial identified an immune classifier, called ImPrintTN, in hopes of identifying which patients with early-stage TNBC may not benefit from immunotherapy. In the 28% of patients who were ImPrintTN+ , 74% of patients achieved a pCR. In patients who were ImPrintTN-, only 16% of patients achieved a pCR [78]. Further validations of this biomarker is needed, but it suggests that a proportion of patients with early-stage TNBC may be able to avoid immunotherapy when it is unlikely to have a benefit.

Neoadjuvant ADCs in early TNBC is an area of active research. The phase II NeoSTAR trial found a pCR rate of 30% with SG monotherapy [79]. The SOLTI TOT-HER3 trial studied neoadjuvant patritumab deruxtecan as a single dose in a window-of-opportunity phase I trial found an ORR of 35% [80].

The Neo-N phase II trial investigated the effect of lead-in vs. concurrent neoadjuvant immunotherapy. Patients with early-stage TNBC were randomized to A) lead-in nivolumab followed by nivolumab with carboplatin and paclitaxel, or B) up-front nivolumab, carboplatin and paclitaxel followed by nivolumab monotherapy. No difference in pCR was seen between the two arms (50.9% vs. 54.5%) [81]. Notably, 66.7% of patients with high TILs and 70.6% of patients with PD-L1 positive disease achieved pCR, delineating a potentially efficacious anthracycline-sparing neoadjuvant regimen [81]. EFS data is pending.

Dual neoadjuvant ICI therapy with combined PD-1 and CTLA-4 agents in early-stage TNBC has been investigated in two phase II trials. The BELLINI trial treated 31 patients with TILs ≥ 5% with neoadjuvant nivolumab ± ipilumumab followed by chemotherapy or surgery. Evidence of immune activation (defined as doubling of CD8 + T cell or IFN-γ) was seen in 58% of patients [82]. Of the three patients who underwent surgery without neoadjuvant chemotherapy, one pCR and one near-pCR was seen. All patients with radiographic response had TILs > 40% [82]. The CHARIOT trial is a phase II, single arm trial of patients with stage III TNBC with RCB  ≥ 15 mm or ≥ 10 mm with node-positive disease after neoadjuvant AC. Patients were treated with neoadjuvant paclitaxel, ipilumumab and nivolumab followed by adjuvant nivolumab. In this high-risk population, overall pCR rates were 24.4%, with pCR rates of 44.4% in the PD-L1 positive subset [83]. Recently presented EFS and OS data showed a remarkable 100% 3-year EFS and OS in the PD-L1 and/or TIL high subset, even though the minority of patients achieved a pCR [83, 84].

Investigations into adjuvant ICIs in TNBC have had limited success. The phase III IMpassion030 / ALEXANDRIA study evaluated the effect of adjuvant atezolizumab with paclitaxel followed by atezolizumab with AC or EC compared with chemotherapy alone in 2199 patients with stage II-III TNBC who underwent upfront surgery. After a median follow up of 25.3 months, the study was halted after a futility analysis found the study was unlikely to meet its primary endpoint of improved iDFS vs. chemotherapy with a HR of 1.12 (0.87–1.45) [85]. When this study was designed, it was unclear if neoadjuvant vs. adjuvant chemotherapy with immunotherapy would provide better outcomes for patients with TNBC. Mounting evidence now points towards focusing on upfront systemic treatment including immunotherapy for early-stage TNBC, with a tailored approach to adjuvant immunotherapy (Table 2).

**Table 2:** Current and upcoming clinical trials of immune checkpoint inhibitors in early triple negative breast cancer

Trial name:	Primary author:	Year:	Study design:	Line of therapy	Stage	# Patients:	Drug regimen	Results
*Pembrolizumab*								
NCT02622074 KEYNOTE-173	Schmid	2020	Phase Ib	Neoadjuvant	cT1c, N1-N2; T2-T4c, N0-N2	60	Nab-Paclitaxel + Pembrolizumab ± Carboplatin then AC	Overall pCR 60% (range 49%–71%) **PD-L1 CPS associated with higher rate of pCR (*p* = 0.0127)** **sTILs associated with higher rate of of pCR (*p* = 0.0085)**
NCT01042379 I-SPY2	Nanda	2020	Phase II	Neoadjuvant	cT2-4d, N0-3	29	Arm A: Paclitaxel + Pembrolizumab then AC → ± adjuvant Pembrolizumab Control: Paclitaxel then AC	pCR 60% (95% CI 44–75) vs 22% (13–30)
NCT01042379 I-SPY2	Liu	2019	Phase II	Neoadjuvant	T ≥ 2.5 cm; HER2 negative	73	Arm A: Paclitaxel + Pembrolizumab Control: Paclitaxel then AC	pCR 21% (95% CI 9–32) vs 20% (15–25)
NCT01042379 I-SPY2	Chien	2021	Phase II	Neoadjuvant	T ≥ 2.5 cm	29	Arm A: Paclitaxel + Pembrolizumab + SD-101 then AC + Pembrolizumab Control: Paclitaxel then AC	pCR 44% vs. 28%
NCT00036488 KEYNOTE-522	Schmid	2020	Phase III	Neoadjuvant + Adjuvant	cT1N1-2, cT2-4, N0-2	1174	Arm A: Carboplatin + Paclitaxel + AC/EC + Pembrolizumab → adjuvant Pembrolizumab Arm B: Carboplatin + Paclitaxel + AC/EC + placebo → adjuvant placebo	**pCR 64.8% (95% CI 59.9–69.9) vs 51.2% (44.1–58.3)** **PD-L1 CPS ≥ 1 pCR 68.9% vs 54.9%** **Risk of Recurrence HR = 0.63 (0.48–0.82)** **5-year EFS 81.2% vs 72.2%; HR 0.63 (0.49–0.81)** **5-year OS 86.6% (84.0–88.8) vs 81.7% (77.5–85.2)**
NCT03639948 NeoPACT	Sharma	2022	Phase II	Neoadjuvant	Stage I-III	117	Carboplatin + Docetaxel + Pembrolizumab	pCR 58% (95% CI: 48–67) 3-year EFS overall 86% EFS pCR subgroup: 98% EFS no pCR subgroup 68%
NCT04373031 NeoIRX	Page	2023	Phase II	Neoadjuvant	Stage II/III	12	Arm A: Pembrolizumab + IRX-2 then AC/T + pembrolizumab Arm B: Pembrolizumab then AC/T + Pembrolizumab	pCR 83% vs. 33%; terminated early due to withdrawal of support for IRX-2
*Atezolizumab*								
NCT03197935 IMpassion031	Mittendorf	2020	Phase III	Neoadjuvant	cT2-4, N0-3	333	Arm A: Atezolizumab + AC + Nab-paclitaxel Arm B: placebo + AC + Nab-paclitaxel	**ITT pCR 57.6% (95% CI 50–65) vs 41.1% (34–49), Difference 17% (6–27)** **PD-L1 ≥ 1 pCR 68.8% (57–79) vs 49.3% (38–61), Difference 20% (4–35)**
NCT002620280 NeoTRIPaPDL1/Michaelangelo	Gianni	2022	Phase III	Neoadjuvant	cT1N1-3; cT2-4d, N0-3	280	Arm A: Atezolizumab + Carboplatin + Nab-paclitxel → adjuvant AC/EC Arm B: Carboplatin + Nab-paclitaxel → adjuvant AC/EC	pCR 48.6% vs. 44.4%, OR 1.18 (95% CI 0.74–1.89) PD-L1 + expression influenced rate of pCR, OR 2.08 (1.64–2.65)
NCI10013	Ademuyiwa	2022	Phase II	Neoadjuvant	cT2-4, N0-3	67	Arm A: Atezolizumab + Carboplatin + Paclitxel Arm B: Carboplatin + Paclitxel	pCR 55.6 vs 18.8%
NCT03498716 IMpassion030/ALEXANDRIA	Ignatiadis	2023	Phase III	Adjuvant	Stage II-III	2199	Arm A: Atezolizumab + ddAC + Paclitaxel Arm B: ddAC + Paclitaxel	iDFS HR 1.12 (95% CI 0.87—1.45) iDFS PD-L1 + 1.03 (0.75—1.42)
*Durvalumab*								
NCT02489448	Foldi	2021	Phase I/II	Neoadjuvant	cT1-3, N0-3	59	Durvalumab + AC + Nab-Paclitaxel	pCR 44% PD-L1 ≥ 1% pCR 55%
NCT02685059 GeparNUEVO	Loibl	2019	Phase II	Neoadjuvant	cT2-4d, N0-3	174	Arm A: Durvalumab window then Nab-Paclitaxel + Durvalumab then EC + Durvalumab Arm B: Placebo window then Nab-Paclitaxel + Placebo then EC + Placebo	pCR 53% vs. 44%, OR 1.45 (95% CI 0.80–2.63) **3-year iDFS 85.6% vs. 77.2%, HR 0.48 (0.24–0.97)** **3-year DDFS 91.7% vs. 78.4%, HR 0.31 (0.13–0.74)** **3-year OS 95.2% vs. 83.5%, HR 0.24 (0.08–0.72)**
NCT01042379 I-SPY2	Pusztai	2021	Phase II	Neoadjuvant	Stage II-III	21	Arm A: Durvalumab + AC + Olaparib + Paclitaxel Arm B: AC + Paclitaxel	pCR 47% vs 27%
*Nivolumab*								
BCT1902 Neo-N	Loi	2023	Phase II	Neoadjuvant	cT1cN1; cT2-4, N0-1	110	Arm A: Nivolumab Lead-In then Nivolumab + Carboplatin + Paclitaxel Arm B: Nivolumab + Carboplatin + Paclitaxel then Nivolumab alone	pCR 50.9% vs. 54.5% sTIL high vs low: 66.7% vs 45.7% PD-L1 positive vs negative: 70.6% vs 33.3%
*Dual Immunotherapy*								
EudraCT: 2018-004188-30 BELLINI	Nederlof	2022	Phase II	Neoadjuvant	Stage I-III, TILs ≥ 5%	31	Arm A: Nivolumab then chemotherapy or surgery Arm B: Nivolumab + Ipilumumab then chemotherapy or surgery	Immune Activation = Doubling CD8+ T-cells or IFN-g seen in 58% of patients. Of 3 patients who went for surgery without neoadjuvant chemo, 1 pCR and 1 near-pCR
BCT1702 CHARIOT	Loi	2022	phase II	Neoadjuvant + Adjuvant	Stage III with ≥ 15 mm RD or 10 mm RCB + one positive lymph node after AC × 4	34	Ipilimumab + Nivolumab + Paclitaxel → adjuvant Nivolumab	pCR 24.2% PD-L1+ pCR 37.5% 3-year EFS 61.3% PD-L1+ 3-year EFS 100% 3-year OS 71.9% PD-L1+ 3-year OS 100%
*Selected Upcoming Clinical Trials*								
NCT05929768 SWOG2212/SCARLET			Phase III	Neoadjuvant	T2-4, N0, M0 or T1-3, N1-2, M0 with high TILs,PD-L1	2400	Arm A: Carboplatin + Paclitaxel + AC + Pembrolizumab → adjuvant Pembrolizumab Arm B: Carboplatin + Docetaxel + Pembrolizumab	EFS
NCT02954874 SWOG1418/NRGBR0006			Phase III	Adjuvant	≥ 1 cm or N+ RCB	1000	Arm A: Pembrolizumab Arm B: Observation	iDFS in 1) all randomized patients and 2) PDL-1+ patients
NCT02926196 A-BRAVE			Phase III	Adjuvant	RCB	335	Arm A: Avelumab Arm B: Observation	DFS
NCT05812807 Optimice-pCR/A012103			Phase III	Adjuvant	achieved pCR	1295	Arm A: Pembrolizumab Arm B: Observation	RFS
NCT03281954 GeparDouze			Phase III	Neoadjuvant + Adjuvant	Stage II-III	1550	Arm A: Atezolizumab + Carboplatin + Paclitaxel then AC or EC → adjuvant Atezolizumab Arm B: Placebo + Carboplatin + Paclitaxel then AC/EC → adjuvant Placebo	pCR EFS
NCT04427293 BRE-03			Phase I	Neoadjuvant Window of Opportunity	Early-stage	12	Lenvatinib + Pembrolizumab	TILs present in biopsy
NCT05973864			Phase III	Adjuvant	Early-stage with RCB	418	Arm A: Capecitabine + Pembrolizumab Arm B: Pembrolizumab	iDFS
NCT03036488			Phase III	Neoadjuvant + Adjuvant	Locally Advanced	1174	Arm A: Pembrolizumab + Chemotherapy → adjuvant Pembrolizumab Arm B: Placebo + Chemotherapy → adjuvant Pembrolizumab	pCR
NCT04335669 NordicTrip			Phase III	Neoadjuvant	Stage II-III	920	Arm A: EC + Pembrolizumab then Carboplatin + Paclitaxel + Pembrolizumab Arm B: EC + Capecitabine + Pembrolizumab then Carboplatin + Paclitaxel + Pembrolizumab	pCR

Results in bold are statistically significant

### Vaccines in TNBC and across subtypes

The majority of breast cancer vaccine clinical trials have focused on the metastatic setting. The Theratrope trial was a phase III clinical trial of 1028 patients with any subtype of mBC who were treated with sialyl-TN, a Muc1 epitope, conjugated to keyhole limpet hemocyanin (KLH) protein vs. KLH alone, with a primary endpoint of time to progression [86]. Patients were given a priming dose of cyclophosphamide 3 days prior to vaccine administration. Though the treatment group did produce anti-mucin antibodies, no difference in time to progression or overall survival was seen [86]. Adagloxad simolenin, a Globo-H epitope conjugated to KLH with cyclophosphamide, was evaluated in a phase II trial of patients with mBC [87]. No difference in overall PFS was seen in comparison to the placebo group, though patients who achieved higher anti-GloboH titers did have an improved PFS [87]. The upcoming phase III GLORIA (NCT03562637) trial will investigate this vaccine in patients with TNBC expressing Globo-H in the adjuvant setting [88].

Virus-based breast cancer vaccines have also been investigated in phase I and II studies, with an objective of infecting antigen-presenting cells to enhance the immunologic response against malignant cells. PANVAC is a poxvirus vaccine that encodes transgenes for Muc-1, CEA, and three co-stimulatory molecules [9]. Phase I trials of PANVAC demonstrated safety and immunoreactivity, with one patient with mBC achieving a complete response [9]. The phase II study combined PANVAC with docetaxel, with a nearly-significant improvement in PFS (7.9 months vs. 3.9 months, HR 0.65, *p* = 0.09) compared to docetaxel alone [89]. While most viral vaccines have focused on heavily pretreated patients, two vaccines have moved into the neoadjuvant space. Pelareorep, a type III reovirus, in combination with paclitaxel, was found to nearly-significantly increase overall survival in the metastatic setting (17.4 months vs. 10.4 months, HR 0.65, *p* = 0.1) [90]. Pelareorep is now being studied in the neoadjuvant setting in early-stage TNBC, with preliminary data demonstrating effective priming of an adaptive immune response [91]. A recent phase II study of neoadjuvant intra-tumoral talimogene iaherparepvec (T-VEC) + paclitaxel followed by AC in stage II-III TNBC found a pCR rate of 45.9%, with a 2-year DFS rate of 89%, with no recurrences in patients with RCB 0 or 1 [92].

### Upcoming clinical trials in TNBC

In the a/m setting, multiple phase III studies are evaluating ADCs with ICI in the first line. For patients with PD-L1+ disease, the upcoming ASCENT-04 (NCT05382286) trial will investigate first-line SG with pembrolizumab vs. treatment of physician’s choice with pembrolizumab for PD-L1+ disease. The TROPION-Breast-05 (NCT06103864) trial will assess Dato-DXd with durvalumab against the KEYNOTE-355 regimen (chemotherapy + pembrolizumab). These studies will determine the optimal first-line therapy for patients with PD-L1+ a/mTNBC. Atezolizumab in combination with ladoratizimab vedotin (LV) is being studied in in the first line for patients with ICI-naïve a/m TNBC in another arm of the phase I/II MORPHEUS trial (NCT03424005).

For patients with PD-L1- a/m TNBC disease, upcoming phase III trials to determine the optimal first-line therapy include the ASCENT-03 (NCT05382299) trial, which is investigating SG compared to treatment of physician’s choice and TROPION-Breast 02 (NCT05374512), which investigates Dato-DXd against treatment of physician’s choice. Phase II studies such as SACI-IO TNBC (NCT04468061) study will assess SG with or without pembrolizumab in the first line while SNGLVA-002 (NCT03310957) trial will investigate first-line LV with pembrolizumab.

Deescalating neoadjuvant chemoimmunotherapy in early TNBC based on high level of TILs is being investigated in the upcoming phase II NeoTRACT (NCT05645380) trial. Patients with TILs ≥ 5% will be treated with carboplatin, docetaxel and pembrolizumab, while patients with low TILs < 5% will receive the standard Keynote-522 regimen.

Several upcoming trials will clarify the role of adjuvant ICIs, with an emphasis on tailoring therapy based on success of neoadjuvant treatment. The Optimice-pCR (NCT05812807) trial is a phase III trial that will clarify whether patients who achieve pCR can be spared adjuvant immunotherapy. Patients with early-stage TNBC who achieved pCR with combination pembrolizumab and chemotherapy will be randomized to adjuvant pembrolizumab vs. observation, with a primary outcome of recurrence-free survival.

For patients with RCB after neoadjuvant therapy, two upcoming phase III trials will evaluate the benefit of one year of adjuvant ICI vs. observation. The SWOG1418 / NRGBR0006 (NCT02954874) will study pembrolizumab in patients with > 1 cm RCB or positive lymph nodes, with co-endpoints of iDFS overall and in a PD-L1+ subgroup. The A-BRAVE (NCT02926196) trial is studying avelumab in either A) patients who underwent upfront surgery followed by adjuvant chemotherapy, or B) patients with RCB after neoadjuvant therapy.

Combinations of ICI with VEGF inhibition is undergoing further investigation, based on the success of the AtractIB study. The BELLA (NCT04739670) phase II trial is investigating atezolizumab, bevacizumab, carboplatin and gemcitabine in patient with early-relapsing PD-L1+ TNBC, while the MARIO-3 (NCT03961698) phase II trial is investigating atezolizumab, bevacizumab, nab-paclitaxel and eganelisib (A PI3Kγ inhibitor) in the first line for a/m TNBC.

Clinical trials evaluating ADCs, with or without ICIs, in the adjuvant setting are also underway. The SASCIA (NCT04595565) phase III trial is investigating adjuvant SG monotherapy in comparison to treatment of physician’s choice of therapy for patients with residual TNBC or HR+ /HER2- disease after neoadjuvant therapy and surgery. Similarly, the ASCENT-05/Optimice-RD (NCT05633654) study is testing SG with pembrolizumab against treatment of physician’s choice in the adjuvant setting. The TROPION-Breast-03 (NCT05629585) trial is investigating adjuvant Dato-Dxd with or without durvalumab versus standard of care therapy for patients with RCB, including both patients who did and did not receive neoadjuvant immunotherapy. These studies will clarify the benefit of adjuvant ICI for patients with RCB-III disease, given the poor outcomes seen in that subgroup in the KEYNOTE-522 study.

In the neoadjuvant setting, the TROPION-Breast-04 (NCT06112379) trial will compare the BEGONIA regimen of Dato-DXd with durvalumab against the KEYNOTE-522 regimen in the neoadjuvant setting for patients with stage II-III TNBC or HER2-low disease, with the hope of de-escalating toxicities of chemotherapy in the curative setting. Cohort 2 of the NeoSTAR (NCT04230109) trial will investigate SG with pembrolizumab in another de-escalated neoadjuvant regimen.

## HR positive, HER2 negative breast cancer

The majority of breast cancers express estrogen and/or progesterone receptors, collectively termed hormone receptor positive (HR+). While HR+ disease has been demonstrated to respond, at least initially, to endocrine therapies, investigations into immunotherapy for the most common subtype of cancer have found less success. This may be explained, in part, by lower expression of PD-L1 and TILs, with only 15% of HR+ breast cancer expressing PD-L1 CPS > 10.[30] There are no FDA-approved immunotherapies in HR + breast cancer to date. Nonetheless, efforts are ongoing to optimize treatment regimens with some encouraging results.

### Advanced and metastatic HR+ /HER2- breast cancer

The first trials of immunotherapy in HR+ /HER2- disease evaluated single-agent pembrolizumab in heavily pretreated, PD-L1 CPS ≥ 1 mBC with an ORR of 12%[93]. Combination chemotherapy and immunotherapy was investigated in a phase II trial of eribulin with or without pembrolizumab in patients who had received 0–2 prior lines of chemotherapy and at least two lines of hormonal therapy, which did not show an ORR, PFS or OS benefit.[94].

Following the success of the anti-TROP-2 ADC SG in TNBC, the TROPiCS-2 trial investigated SG vs treatment of physician’s choice in patients with endocrine-resistant HR+ /HER2- MBC in the third or later line. Patients who received SG had a significantly improved PFS (5.5 vs 4.0 months) [95] and OS (14.1 vs 11.2 months) [96], which led to FDA approval of SG in the third or later line of HR+ /HER2- MBC in February 2023 [95].

The SACI-IO HR+ phase II trial investigated SG with or without pembrolizumab in HR+ /HER2- mBC with any PD-L1 status after ≥ 1 endocrine therapy, 0–1 lines of chemotherapy and no prior immunotherapy or ADC in the metastatic setting. No PFS benefit was seen in the combination therapy arm (8.1 months vs. 6.2 months, HR 0.81) with immature OS data suggesting no difference at 12.5 months median follow-up [97]. In the PD-L1 CPS ≥ 1 subgroup, a non-significant 4.4 month increase in PFS was seen with combination SG and pembrolizumab, though OS data is immature [97].

Studies of Dato-DXd in HR+ /HER2- MBC have also shown promising results. The HR+ /HER2- arm of the phase I Pan-Tumour 01 study found an ORR of 26.8% in heavily pretreated patients who received Dato-DXd monotherapy, with a PFS of 8.3 months [56]. The phase III TROPION-Breast 01 trial compared Dato-DXd to physician’s choice of chemotherapy in the second or third line, with an improved PFS of 6.9 months versus 4.9 months (HR 0.63) but no demonstrated overall survival benefit according to a press release [98, 99]. Other ADCs in this space include enfortumab vedotin, which found an ORR of 15.6% with a PFS of 5.4 months in heavily pretreated patients in the phase II EV-202 study [58], as well as patritumab deruxtecan, which found a 3-months RR of 28.6% in the second line in the phase II ICARUS-BREAST-01 trial [100].

The AIPAC study is investigating LAG-3 modulation in breast cancer. This phase II trial randomized patients with HR+ /HER2- mBC that has developed resistance to endocrine therapy to paclitaxel with eftilagimod alpha, a LAG-3 inhibitor, or paclitaxel alone. While there was no significant difference in PFS or OS overall, patients younger than 65 did have a significant 7-month OS benefit, and patients with increased CD8 count 6 months after treatment had significantly improved OS [4].

Given the demonstrated benefit of CDK4/6 inhibition in HR+ disease, trials investigating CDK4/6 inhibitors (CDK4/6i) in combination with ICIs are of interest but have been met with safety concerns. A phase 1b study of abemaciclib with pembrolizumab with or without anastrozole in HR+ /HER2- mBC previously untreated with a CDK4/6i found an ORR of 23% in the first line and an ORR of 29% of patients who had previously received chemotherapy [101]. Rates of grade 3 adverse effects were seen in 69.2% in the untreated group and 60.7% in the previously treated group, with one case of grade-5 interstitial lung disease (ILD) in the first-line setting and higher than expected hepatotoxicity. The NEWFLAME phase II study evaluated nivolumab with abemaciclib and letrozole or fulvestrant in the first or second line of HR+ /HER2- mBC. Though the first 17 patients enrolled experienced an ORR of 54.5% in the letrozole arm and 40.0% in the fulvestrant arm, the trial was stopped early for safety [102]. Over 90% of patients experienced a grade 3 adverse effect, with one ILD-induced grade 5 event in the letrozole arm. Similarly, the Checkmate 7A8 trial of neoadjuvant nivolumab, palbociclib and anastrozole was stopped after 43% of patients discontinued treatment due to adverse events, including hepatotoxicity, neutropenia, rash and ILD [103]. The PACE phase II study of fulvestrant, fulvestrant with palbociclib, or fulvestrant with palbociclib and avelumab was studied in patients with HR + /HER2- mBC who had progressed on prior CDK4/6i and aromatase inhibitor. While it was designed to evaluate the efficacy of continuing CDK4/6i after progression, a non-significant 3.3-month PFS benefit was seen in the fulvestrant with palbociclib and avelumab arm in comparison to fulvestrant alone (HR 0.75) [104]. Grade 3 or 4 adverse events were rare, with no ILD as seen in the above trials with pembrolizumab and nivolumab.

### Early-stage HR + /HER2- breast cancer

Though HR + breast cancer overall is associated with a good prognosis, treatment escalation with chemotherapy is indicated for patients with high risk of recurrence. The role for immunotherapy in combination with chemotherapy for certain high-risk subgroups is of active interest. Data from I-SPY-2 showed an improved rate of pCR with pembrolizumab concurrent with paclitaxel followed by doxorubicin and cyclophosphamide (T-AC) (pCR 30% vs. 13%) in HR + /HER2-, MammaPrint high-risk breast cancer with tumor size ≥ 2.5 cm [64]. In the phase III KEYNOTE-756 trial, the benefit of neoadjuvant and adjuvant pembrolizumab in grade 3, high-risk ER+ disease was clarified by comparing the I-SPY-2 regimen to standard T-AC in the neoadjuvant setting, followed by adjuvant endocrine therapy with pembrolizumab or placebo in the adjuvant setting. Seventy-six percent of patients were PD-L1 positive. An 8.5% improvement in pCR rates was seen in the pembrolizumab arm (24.3% vs. 15.6%, *p* = 0.00005) with a larger benefit seen in patients with node-positive disease, PD-L1 positivity as defined by CPS ≥ 1 (29.7% vs. 19.6%), and ER positivity < 10% [105]. Of note, in this trial clinicians were given the option of every-2-week dosing or every-3-week dosing. EFS data is pending to evaluate the benefit of adjuvant pembrolizumab.

Nivolumab in the neoadjuvant setting has also found success. The GIADA trial evaluated patients with stage II-III luminal B breast cancer, finding a pCR rate of 16.3% after therapy with EC followed by nivolumab, troptorelin and exemestane [106]. The phase III Checkmate 7Fl trial aimed to investigate neoadjuvant and adjuvant nivolumab in 1278 patients with high-risk ER+ /HER2- breast cancer. Patients were randomized to neoadjuvant paclitaxel with or without nivolumab followed by AC, followed by adjuvant endocrine therapy and nivolumab or placebo. A significant pCR advantage (24.5% vs. 13.8%) was seen, with an enhanced advantage in the 35% of patients who were PD-L1+ (44.3% vs. 20.2%). A pCR benefit was also seen in patients with ER < 50%, PR < 10%, and TILS ≥ 1%. This trial confirmed a pCR benefit with immunotherapy in ER+ disease [107]. However, the adjuvant portion of the study stopped enrollment early after adjuvant abemaciclib gained FDA approval based on data from MonarchE because CDK4/6i cannot be safely combined with ICIs due to safety concerns as discussed above. EFS data is pending.

Data from I-SPY2 also suggests that neoadjuvant durvalumab in combination with paclitaxel and olaparib may benefit patients with stage II-III HR+ /HER2- breast cancer. Patients who were high risk for recurrence by MammaPrint (either High-1 or High-2) were included. While no difference in pCR rate was seen in the high 1 group, 64% of patients with high 2 disease achieved a pCR with immunotherapy, compared to 22%s in the paclitaxel control group [76]. This finding suggests that MammaPrint High-2 could be a predictive biomarker for immunotherapy in HR+ /HER2- early breast cancer, though further validation is needed.

ADC in combination with ICI in early-stage HR+ /HER2- breast cancer has found early promising results. Neoadjuvant Dato-DXd with durvalumab for high-risk, early stage HR+ /HER2- breast cancer achieved an overall pCR rate of 50%, with rates of 79% in the immune signature subtype [108] (Table 3).

**Table 3:** Current and upcoming clinical trials of immune checkpoint inhibitors in hormone receptor positive breast cancer

Trial name	Primary author	Year	Study design	Line of therapy	Stage	# Patients	Drug regimen	Results
*Pembrolizumab*								
NCT02054806 KEYNOTE-028	Rugo	2018	Phase Ib	Heavily Pretreated	Advanced, PD-L1 CPS ≥ 1	25	Pembrolizumab	ORR 12%
NCT03222856 KELLY	Perez-Garcia	2021	Phase II	> First Line	Advanced	44	Eribulin + Pembrolizumab	CBR 56.8% (95% CI 41.0–71.7) ORR 40.9% (26.3–56.8)
NCT03051659	Tolaney	2020	Phase II	Two or more lines of hormonal therapy; 0–2 lines of chemotherapy	HR+ /HER2- MBC	88	Arm A: Eribulin + Pembrolizumab Arm B: Eribulin	ORR 27% (95% CI 14.9–42.8) vs 34% (20.5–39.9) PFS 4.1 (3.5–6.2) vs 4.2 mo (3.7–6.1), HR 0.80 (0.50–1.26) OS 13.4 (10.4-NE) vs. 12.5 mo (8.6-NE), HR 0.87 (0.48–1.59)
NCT03044730	Shah	2020	Phase II	Median 1 prior therapy	Metastatic	14	Capecitabine + Pembrolizumab	ORR 14%; PFS 5.1 mo; OS not reached
NCT01042379 I-SPY2	Nanda	2020	Phase II	Neoadjuvant	cT2-4d, cN0-3 HR+ /HER2-	40	Arm A: Weekly paclitaxel + Pembrolizumab followed by AC Arm B: Weekly paclitaxel + placebo followed by AC	pCR 30% (95% CI: 17–43) vs. 13% (CI 7–19)
NCT02395627	Terranova-Barberio	2020	Phase II	Heavily pretreated, Metastatic ER+ , PD-L1 negative	Metastatic	34	Arm A: Tamoxifen + Vorinostat + Pembrolizumab (C1) Arm B: Tamoxifen + Vorinostat + Pembrolizumab (C2)	ORR 3.7% CBR 18.5% Stopped early for lack of efficacy
NCT03725059 KEYNOTE-756	Cardoso	2023	Phase III	Neoadjuvant & adjuvant	T1c-2, cN1-2 or T3-4, cN0-2; grade 3	1278	Arm A: Pembrolizumab + Paclitaxel then Doxorubicin + Cyclophosphamide → Adjuvant Pembrolizuamb + Endocrine Therapy Arm B: Placebo + Paclitaxel then Doxorubicin + Cyclophosphamide → Adjuvant Placebo + Endocrine Therapy	**pCR 24.3% (95% CI: 21.0–27.8) vs. 15.6% (12.8–18.6) (*p* = .00005)** Stage II disease pCR 25.8% vs 16.7% Stage III disease pCR 21.6% vs. 13.6% N positive pCR 25.1% vs. 15.8% N negative pCR 16.9% vs. 13.1% PD-L1 + pCR 29.7% vs 19.6% PD-L1 + , ER + < 10% pCR 57.6% vs. PD-L1 + , ER > 10% 33.3% EFS immature
*Atezolizumab*								
NCT03147040 GELATO	Voorwerk	2023	Phase II	First or second line	Metastatic, HER2- Lobular	23 (18 with ER+ disease, 5 with TNBC)	Carboplatin + Atezolizumab	ORR 17%; CBR 26% 4 of 6 patienst with clinical benefit had TNBC
*Tremelimumab*								
	Vonderhiede	2010	Phase I	> First line	Metastatic	26	Tremelimumab + Exemestane	Stable disease in 42% at 12 weeks
	Santa Maria	2018	Pilot study	> First line	Metastatic	11	Tremelimumab + Durvalumab	ORR 0%
*Durvalumab*								
NCT02811497 METADUR	Taylor	2020	Phase II	> First line ER+ breast cancer		9	Arm A: Azacitazine + Durvalumab Arm B: Azacitazine + Durvalumab + vitamin C	no response
NCT02734004 MEDIOLA	Domchek	2020	Phase I/II	> Third line	Metastatic	34	Olaparib + Durvalumab	Tolerable Safety DCR at 12 weeks 80% (90% CI: 64.3–90.9)
NCT01042379 I-SPY2	Pusztai	2021	Phase II	Neoadjuvant	Stage II-III HR+ /HER2-; MammaPrint high-risk	65	Arm A: Paclitaxel + Durvalumab + Olaparib Arm B: Paclitaxel	pCR 28% (95% CI 18–38) vs. 14% (9–19); MammaPrint MP1 pCR 9% (0–18) vs. 10% (5–18) MammaPrint MP2 pCR 64% (47—80) vs. 22% (13—32)
NCT03875573 Neo-CheckRay	De Caluwe	2024	Phase II	Neoadjuvant	Luminal B, Mammaprint High-Risk	135	Arm A: AC + paclitaxel followed by preoperative radiation Arm A: AC + paclitaxel + durvalumab followed by preoperative radiation Arm C: AC + paclitaxel + durvalumab + oleclumab followed by preoperative radiation	pCR 17.8% (95% CI 6.6–28.9) vs 31.8% (18.1–45.6) vs 35.6% (21.6–49.5)
*Nivolumab*								
NCT04659551 GIADA	Dieci	2021	Phase II	Neoadjuvant	Stage II-IIIA, HR+ , HER2-	43	EC followed by Nivolumab + Troptorelin + Exemestane	pCR 16.3% (95% CI: 7.4—34.9) **PAM50 basal pCR 50% vs. Luminal A pCR 9% vs. Luminal B 8% (*p* = 0.017)**
WJOG9917B NEWBEAT	Ozaki	2022	Phase II	First line	Metastatic HR+ /HER2-	17	Bevacizumab + Nivolumab + Paclitaxel	ORR 74% PFS 16.1 months
NCT04109066 CheckMate 7FL	Loi	2023	Phase III	Neoadjuvant	Stage T1c-2, N1-2 or T3-4, N0-2	521	Arm A: Nivolumab + Paclitaxel then Doxorubicin + Cyclophosphamide → Adjuvant Endocrine Therapy Arm B: Placebo + Paclitaxel then Doxorubicin + Cyclophosphamide → Adjuvant Endocrine Therapy	**pCR 24.5% (95% CI 19.4–30.2) vs. 13.8% (9.8–13.7), Difference 10.5 (4.0–16.9)** **PD-L1 + : pCR 44.3% (33.7–55.3) vs. 20.2% (12.3–30.4), Difference 24.1 (10.7–37.5)** PD-L1-: pCR 14.2% vs. 10.7%
*Avelumab*								
NCT03147287 PACE	Mayer	2024	Phase II	> First line ER+ breast cancer	Metastatic	220	Arm A: Fulvestrant Arm B: Fulvestrant + Palbociclib Arm C: Fulvestrant + Palbociclib + Avelumab	PFS Arm A vs Arm B: 4.8 (90% CI 2.1–8.2) vs. 4.6 mo (3.6–5.9), HR 1.11 (0.79–1.55) PFS Arm A vs Arm C: 4.8 (2.1–8.2) vs 8.1 mo (3.2–10.7), HR 0.75 (0.50–1.12) ORR Arm A 7.3% (1.5–13.0), Arm B 9.0 (4.5–13.5), Arm C 13.0 (5.4–20.5) CBR Arm A 29.1 (19.0–39.2), Arm B 32.4 (25.1–39.7), Arm C 35.2 (24.5–45.9)
*Combination with CDK4/6 inhibitors*								
WJOG11418B NEWFLAME	Masuda	2022	Phase II	First or second line	Metastatic	17	Cohort 1: Nivolumab + Abemaciclib + Fulvestrant Cohort 2: Nivolumab + Abemaciclib + Letrozole	ORR 54.5% (95% CI 28.0–78.7) vs. 40.0% (11.7–76.9) Safety: Grade ≥ 3 AE: 92% vs. 100% (neutropenia, hepatotoxicity, ILD) Early termination for safety
NCT04075604 CheckMate 7A8	Jerusalem	2022	Phase Ib/II	Neoadjuvant	T ≥ 2 cm, ER+ /HER2-	21	Cohort 1: Nivolumab + Palbociclib + Anastrazole	43% treatment discontinuation due to AE (hepatotoxicity, neuropenia, rash, ILD) Early termination for safety
NCT02779751	Rugo	2022	Phase Ib	Any	Metastatic	28	Cohort 1: Abemaciclib + Pembrolizumab + Anastrazole Cohort 2: Abemaciclib + Pembrolizumab	ORR 23.1% (95% CI 9.0–43.7) vs. 28.6% (13.2–48.7) DCR 84.6% (65.1–95.6) vs. 82.1% (63.1–93.9) Safety: High rates of grade 3 neutropenia, hepatotoxicity, and diarrhea. 2 grade 5 events in cohort 1
*LAG-3*								
NCT02614833 AIPAC	Wildiers	2024	Phase IIb	HR+ , HER2- MBC	Metastatic, ET-resistant	226	Arm A: Paclitaxel + Eftilagimod Alpha Arm B: Paclitaxel + Placebo	PFS 7.3 (95% CI 6.6–7.5) vs. 7.3 mo (5.5–7.5) OS 20.4 (14.3–25.1) vs. 17.5 mo (12.9–21.8), HR 0.88 (0.64–1.19) Age < 65, OS 22.3 mo (15.3–29.6) vs 14.8 (10.9–18.5), HR 0.66 (0.45–0.97)
*Selected Upcoming Clinical Trials*								
NCT06058377 SWOG2206			Phase III	Neoadjuvant	Stage II/III ER+ /HER2-, MP2/High-2	3680	Arm A: Durvalumab plus AC/T—→ Adjuvant ET Arm B: ACT → Adjuvant ET	pCR iDFS
NCT05747794 AIPAC 3			Phase III	First line	Metastatic, endocrine-resistant HR+ /HER2- or TNBC	771	Arm A: Paclitaxel + Eftilagimod Alpha Arm B: Paclitaxel + Placebo	OS
NCT05159778			Phase I/II	Prior CDK4/6, < 2 chemotherapies, no prior ICI	Metastatic, ET-resistant	47	Odetiglucan + Pembrolizumab	ORR
NCT04895358 KEYNOTE-B49			Phase III	Previously treated	Advanced, PD-L1+	800	Arm A: Pembrolizumab + Chemotherapy Arm B: Placebo + Chemotherapy	PFS in patients with CPS ≥ 10

Results in bold are statistically significant

Overall, there may be a benefit for immunotherapy in early-stage HR+ /HER2- disease, though additional investigation of biomarkers to predict response to immunotherapy is needed given alternative treatment options in this setting.

### Upcoming clinical trials in HR + /HER2- breast cancer

The upcoming SWOG S2206 (NCT06058377) phase III trial will clarify the role for neoadjuvant immunotherapy without adjuvant immunotherapy in patients with ER+ /HER2- MammaPrint High-2 disease, who will receive either durvalumab with AC-T neoadjuvant chemotherapy or AC-T alone. LAG-3 inhibition is under further investigation in the phase III trial AIPAC-003 (NCT05747794), which will investigate paclitaxel with or without eftilagimod alpha in patients with endocrine-resistant, HR+ /HER2- mBC or TNBC not eligible for PD-L1 therapy. Adjuvant ADC therapy with SG vs chemotherapy for HR+ /HER2- residual disease is being investigated in the upcoming SASCIA trial (NCT04595565), while SG with or without pembrolizumab is being evaluated in the first or second-line metastatic setting (NCT04448886).

## HER2 positive breast cancer

HER2 positivity, defined as IHC 3+ , is seen in approximately 20% of breast cancer, though the majority of breast cancer express HER2 to some degree [109]. HER2+ breast cancer has higher TILs, TMB, and PD-L1 expression than HER2- disease, which may correlate with an enhanced response to immunotherapy [110]. To date, no ICI has improved outcomes in comparison to standard HER2-targeted regimens in a randomized clinical trial, though early results may suggest an improvement in PD-L1 positive disease.

### Advanced and metastatic HR-/HER2 + breast cancer

The PANACEA phase Ib/II trial evaluated combination trastuzumab and pembrolizumab in patients with a/m HER2+ breast cancer who had previous progression on trastuzumab. The primary endpoint of ORR in PD-L1+ disease was 15%, with no responses in the PD-L1 negative group [111]. Median PFS did not differ by PD-L1 status (2.7 months vs. 2.5 months), though 12-month OS was numerically higher in the PD-L1+ group (65% vs. 12%) [111]. T-DM1,an ADC which consists of trastuzumab linked to DM1, a cytotoxic microtubule inhibitor, is standard therapy for patients with HER2+ residual disease after neoadjuvant chemotherapy [112].

Pembrolizumab in combination with T-DM1 was investigated in a phase Ib trial in metastatic HER2+ disease, with an ORR of 20% [113]. Atezolizumab in combination with T-DM1 in a/m HER2+ disease was associated with an ORR of 35%, while atezolizumab with trastuzumab, pertuzumab and docetaxel had an ORR of 100% in a phase Ib study [114]. The KATE2 phase II study compared T-DM1 with and without atezolizumab in a/m disease after the first line with no significant improvement in PFS in the ITT analysis, though patients in the treatment arm experienced increased toxicity requiring early unblinding of the treatment arms. However, an exploratory analysis found a trend towards improvement in PFS in patients with PD-L1+ disease [115].

Trastuzumab deruxtecan (T-DXd), a HER2-targeted ADC, has changed the treatment landscape for metastatic breast cancer that expresses HER2 and redefined classification of HER2 expression [109]. The landmark DESTINY-Breast 03 trial showed a dramatic improvement in PFS (29 vs 7.2 months) and OS (39.2 vs 26.5 months) with T-DXd vs T-DM1 in the second-line, with 21.1% of patients in the T-DXd group experiencing a complete response [116–118]. An exploratory subgroup of patients with brain metastasis found an intracranial ORR of 65.7% compared to 34.3% [119]. T-DXd is approved for HER2+ MBC in the second line.

Based on these studies, in addition to studies of T-DXd in other solid tumors such as lung and colorectal cancer, T-DXd was recently granted universal FDA approval for any HER2+ a/m solid cancer after one prior treatment [120–122].

Other ADCs under investigation in HER2+ MBC include trastuzumab duocarmazine (T-Duo), which was compared to chemotherapy in the third or greater line, or pretreated with T-DM1, for patients with HER2+ MBC in the phase III TULIP trial [123]. An improvement in PFS (7.0 vs. 4.9 months, HR 0.63) and a trend towards improved OS (21.0 vs 19.5 months, HR 0.87 (95% CI 0.68–1.12) was seen.[123].

### Advanced and metastatic HER2 low breast cancer

T-DXd was also found to have significant activity in patients who were not traditionally considered to be HER2+ (IHC 3 +) [109, 124, 125]. This redefined the spectrum of HER2 expression from a binary (positive or negative) to a spectrum including HER2-low (IHC 1+ or 2+ , in-situ-hybridization (ISH) negative) and HER2-ultralow (faint, incomplete membrane staining in $\ge$ 10% of tumor cells, less than IHC 1+) [124–126]. The DESTINY-Breast 04 phase II trial found T-DXd to have significantly improved PFS (9.9 vs 5.1 months) and OS (23.4 vs 16.8 months) in comparison to physician’s choice of chemotherapy for HER2-low MBC in the second or third line, leading to FDA approval in this setting [127]. Recent results from DESTINY-Breast 06, which evaluated T-DXd in patients without prior chemotherapy for HER2-low or HER2-ultralow MBC, found a 5-month PFS advantage (HR 0.63) in comparison to standard chemotherapy [126]. T-DXd in combination with durvalumab in patients with HER2-low advanced or MBC was investigated in arm 6 of the BEGONIA trial, which found an ORR of 57% with mPFS 12.6 months [128].

###  Early Stage HR-/HER2+ Breast Cancer

A phase II trial of neoadjuvant chemotherapy followed by pembrolizumab, trastuzumab, and pertuzumab found a pCR rate of 46% [129]. The phase III trial IMPassion050 investigated atezolizumab or placebo with neoadjuvant AC followed by paclitaxel, trastuzumab and pertuzumab, followed by adjuvant atezolizumab or placebo in 454 patients with high-risk HER2+ disease. No difference in pCR was seen in the ITT or PD-L1+ subgroup, with EFS data immature [130]. Adverse events were more common in the treatment group, with two deaths in the immunotherapy arm (alveolitis, septic shock) attributed to the treatment (Tables 4 and 5).

**Table 4:** Current and upcoming clinical trials of immune checkpoint inhibitors in HER2 positive breast cancer

Trial name	Primary author	Year:	Study design	Line of therapy	Setting	Biomarker	# Patients	Drug regimen	Results
*Pembrolizumab*									
NCT02129556 PANACEA	Loi	2019	Phase Ib/II	> First line	Advanced/metastatic	Any	52	Pembrolizumab + Trastuzumab	PD-L1 + RR 15% (90% CI 7–29) PD-L1 + PFS 2.7 mo (2.6–4.0) 12-mo PD-L1 + OS 65% (50–76) PD-L1- PFS 2.5 mo (1.4–2.7) 12-mo PD-L1- OS 12% (CI 1–36)
NCT03988036 KEYRICHED-1	Kuemmel	2021	Phase II	Neoadjuvant	Early HER2+ breast cancer s/p anthracycline	Any	48	Pembrolizumab + Pertuzumab + Trastuzumab	pCR 46% (95% CI 0.31–0.62)
*Atezolizumab*									
NCT04759248 SOLTI-ATREZZO	Ciruelos	2023	Phase Ib	Third line after trastuzumab and anti-HER2 ADC	Advanced/Metastatic	Any	19	Atezolizumab + Trastuzumab + Vinorelbine	ORR 31.6% (95% CI 12.6–56.6) Phase II pending
NCT03595592 APTneo Michaelangelo	Gianni	2023	Phase III	Neoadjuvant	ER+ /HER2+ or ER-/HER2+	Any	661	Arm A: Carboplatin + Paclitaxel + Pertuzumab + Trastuzumab + Placebo Arm B1: Atezolizumab + AC followed by Carboplatin + Paclitaxel + Pertuzumab + Trastuzumab Arm B2: Atezolizumab + Carboplatin + Paclitaxel + Pertuzumab + Trastuzumab	pCR Arm A 57.8% vs Arm B 52.9 (HR 1.33, 95% CI 0.95–1.86, *p* = 0.091) Arm B1 vs B2 pCR 61.9% vs 53.6% (HR 1.402, 95% CI 0.95–2.07, *p* = 0.89) Arm B1 vs. Arm A pCR benefit 9.9% (HR 1.58, 95% CI 1.07–2.33, *p* = 0.022) EFS and OS pending
NCT03726879 IMpassion050	Huober	2022	Phase III	Neoadjuvant and adjuvant	T2-4, N1-3	Any	454	Arm A: Atezolizumab + AC followed by Paclitaxel + Pertuzumab + Trastuzumab → Adjuvant Atezolizumab + Pertuzumab + Trastuzumab or T-DM1 Arm B: Arm A: Placebo + AC then Paclitaxel + Pertuzumab + Trastuzumab → Adjuvant Placebo + Pertuzumab + Trastuzumab or T-DM1	pCR 62.7% vs 62.4%; difference = -.33% (95% CI -9.23–8.57) PD-L1 + pCR 72.5% vs. 64.2%, difference -8.26% (-20.56–4.04)
*Durvalumab*									
NCT02649686 CCTG IND 229	Chia	2019	Phase Ib	> First line	Metastatic	PD-L1 negative	15	Durvalumab + Trastuzumab	ORR 0%
*Selected Upcoming Clinical Trials*									
NCT03125928			Phase II	First line	Advanced/metastatic	Any	50	Atezolizumab + Paclitaxel + Pertuzumab + Trastuzumab	Safety
NCT03199885 NRG-BR004			Phase III	First line	Metastatic	Any	600	Arm A: Atezolizumab + Paclitaxel + Pertuzumab + Trastuzumab Arm B: Placebo + Paclitaxel + Pertuzumab + Trastuzumab	PFS
NCT04759248 SOLTI-ATREZZO			Phase II	Third line after trastuzumab and anti-HER2 ADC	Advanced/metastatic	Any	55	Atezolizumab + Trastuzumab + Vinorelbine	ORR

**Table 5:** Current and upcoming clinical trials of antibody drug conjugates in breast cancer

Trial name	Primary author	Year:	Study design	Line of therapy	Setting	Biomarker:	# Patients	Drug regimen	Results
*Sacituzumab govitecan (SG)*									
NCT01631552 IMMU-132-01	Bardia	2019	Phase I/II	Previously treated	mTNBC		108	SG	ORR 33.3% (95% CI 24.6–43.1) DOR 7.7 mo (4.9–10.8) PFS 5.6 mo (4.1–6.3) OS 13 mo (11.2–13.7)
NCT02574455 ASCENT	Bardia	2021	Phase III	> First line	a/mTNBC		468	Arm A: SG Arm B: physician's choice	**PFS 4.8 vs 1.7 mo, HR 0.41 (95% CI 0.33–0.52)** **OS 11.8 vs 6.9 mo, HR 0.51 (0.42–0.63)** ORR 35% vs 5%
NCT03901339 TROPiCS-02	Rugo	2022	Phase III	Previously treated	HR+ /HER2- MBC		543	Arm A: SG Arm B: physician's choice	**PFS 5.5 vs 4.0 mo, HR 0.66 (95% CI 0.53–0.83)** **OS 14.4 vs 11.2 mo, HR 0.79 (0.65–0.96)**
NCT04448886 SACI-IO HR+	Garrido-Castro	2024	Phase II	Previously treated	HR+ /HER2- MBC		110	Arm A: SG + Pembrolizumab Arm B: SG	PFS 8.4 vs. 6.2 mo, HR 0.76 (95% CI 0.47–1.23) PD-L1 + PFS 11.05 vs 6.68 mo, HR 0.62 (0.29–1.36) OS data immature
NCT03424005 MORPHEUS	Schmid	2023	Phase Ib/II	First line	Advanced/Metastatic	CD8 IHC ≥ 10%	42	Arm A: SG + Atezolizumab Arm B: Nab-Paclitaxel + Atezolizumab	ORR 76.7% (95% CI 57.8–90.1) vs. 66.7% (29.9–92.5) CBR 83.3% (65.3–94.4) vs. 66.7% (29.9–92.5) PFS 12.2 mo vs. 5.9 mo, HR 0.27 (0.11–0.70)*
NCT04230109 NeoSTAR	Spring	2024	Phase II	Neoadjuvant	TNBC		50	Cohort 1: SG	pCR 30% (95% CI 18%-45%) ORR 64% (77&-98%) Higher KI-67 and TILs predictive of pCR with SG
*Trastuzumab emtansine (T-DM1)*									
NCT01196052	Krop	2012	Phase II	Heavily pretreated	HER2 + MBC		110	T-DM1	ORR 34.5% (95% CI: 26.1–43.9) CBR 48.2% (38.8- 57.9) mPFS 6.9 mo (4.2–8.4) mDOR 7.2 mo (4.6-NE)
NCT00509769	Burris	2011	Phase II	Previously treated	HER2 + MBC		112	T-DM1	ORR 25.9% (95% CI: 18.4–34.4) mPFS 4.6 mo (3.9–8.6)
NCT03032107	Waks	2022	Phase Ib	> First Line	HER2 + MBC		20	Pembrolizumab + T-DM1	ORR 20% (95% CI 5.7–43.7); PFS 9.6 mo (2.8–16.0)
NCT02605915 GO29381	Hamilton	2021	Phase Ib	Any	HER2 + MBC		73	Arm A: Atezolizumab + Trastuzumab + Pertuzumab Arm B: Atezolizumab + T-DM1 Arm C: Atezolizumab + Trastuzumab, Pertuzumab, and Docetaxel	Arm A: ORR 33% Arm B: ORR 35% Arm C: ORR 100%
NCT02924883 KATE2	Emens	2020	Phase II	> First line	HER2 + MBC		202	Arm A: T-DM1 + Atezolizumab Arm B: T-DM1 + Placebo	PFS 8.2 (95% CI 5.8–10.7) vs 6.8 mo (4.0–11.1), HR 0.82 (0.55–1.23) PD-L1 + PFS 8.5 mo (5.7-NE) vs. 4.1 mo (2.7–11.1), HR 0.60, (0.32–1.11) Unblinded early for futility and safety in atezolizumab arm
NCT00679341	Hurvitz	2011	Phase II	First line	HER2 + MBC		137	Arm A: trastuzumab + docetaxel Arm B: T-DM1	**PFS 9.2 vs 14.2 mo, HR 0.59 (95% CI: 0.36–0.97)** ORR 58.0% vs 64.2%
NCT00829166 EMILIA	Verma	2012	Phase III	> First line	HER2 + MBC		991	Arm A: T-DM1 Arm B: Lapatinib and Capecitabine	**ORR 43.6% vs 30.8%** **PFS 9.6 vs 6.4 mo, HR 0.65 (95% CI 0.55–0.77)** **OS 29.9 vs 25.9 mo, HR 0.75 (0.64–0.88)**
NCT01120184 MARIANNE	Perez	2017	Phase III	First line	HER2 + MBC		1095	Arm A: Taxane + Trastuzumab Arm B: T-DM1 + Placebo Arm C: T-DM1 + Pertuzumab	PFS: 13.7 vs 14.1 vs 15.2 mo PFS T-DM1 vs control: HR 0.91 (95% CI 0.73–1.13) PFS T-DM1 + pertuzumab vs control: HR 0.87 (0.69–1.08) ORR: 67.9% vs 59.7% vs 64.2%
NCT01419197 THE3RESA	Krop	2014		Third line	HER2 + MBC		602	Arm A: T-DM1 Arm B: treatment of physician's choice	**PFS 6.2 vs 3.3 mo, HR 0.528 (95% CI 0.422–0.661)** **OS 22.7 vs 15.8 mo, HR 0.68 (0.54–0.85)**
NCT01772472 KATHERINE	Von Minckwitz	2019	Phase III	Residual disease	Adjuvant HER2 +		1486	Arm A: T-DM1 Arm B: Trastuzumab	**3-year iDFS 88.3% vs 77.0%, HR 0.50 (95% CI 0.39–0.64)** **7-year iDFS 80.8% vs 67.1%** **7-year OS 89.1% vs 84.4%**
*Trastuzumab deruxtecan (T-DXd)*									
NCT02564900	Modi	2020	Phase I	Heavily pretreated	HER2 low MBC		54	T-DXd	ORR 37.0% (95% CI 24.3–51.3) DOR 10.4 mo (8.8-NE)
NCT03248492 DESTINY-Breast 01	Modi	2020	Phase II	Heavily pretreated	HER2 + MBC		184	T-DXd	ORR 62.0% (95% CI 54.9–69) PFS 19.4 mo (14.5–21) OS 29.1 (24.6–36.1)
NCT03523585 DESTINY-Breast 02	André	2023	Phase III	Third line	HER2 + MBC		608	Arm A: T-DXd Arm B: physician's choice of capecitabine with lapatinib or trastuzumab	**PFS 17.8 vs 6.9 mo,, HR 0.36 (95% CI 0.28–0.45)** **OS 39.2 vs 26.5 mo, HR 0.55 (0.50–0.86)**
NCT03529110 DESTINY-Breast 03	Cortés Hurvitz	2022; 2024 2023	Phase III	> First line	HER2 + MBC		524	Arm A: T-DXd Arm B: T-DM1	**PFS 29.0 vs 7.2 mo, HR 0.30 (95% CI 0.24–0.38)** **OS 52.6 vs 42.7 mo, HR 0.73 (0.56–0.94)**
NCT03734029 DESTINY-Breast 04	Modi	2022	Phase III	> First line	HER2 low MBC		557	Arm A: T-DXd Arm B: physician's choice of capecitabine, eribulin, gemcitabine, paclitaxel, or nab-paclitaxel	**PFS 9.9 vs 5.1 mo, HR 0.50 (95% CI 0.40–0.63)** **OS 23.4 vs 16.8 mo, HR 0.64 (0.49–0.84)**
NCT04494425 DESTINY-Breast 06	Curgliano	2024	Phase III	Previously treated	HR+ /HER2 low or ultralow MBC		866	Arm A: T-DXd Arm B: physician's choice of capecitabine or paclitaxel or nab-paclitaxel	**PFS HER2 low 13.2 vs 8.1 mo, HR 0.62 (95% CI 0.51–0.74)** PFS HER2 ultralow 13.2 vs 8.3 mo, HR 0.78 (0.50–1.21) **Overall PFS 13.2 vs 8.1 mo, HR 0.63 (0.53–0.75)**
NCT03523572	Hamilton	2021	Phase Ib	Previously treated	Metastatic HER2+ disease that progressed on T-DM1 or HER2 low that progressed on prior treatment		52	Nivolumab + T-DXd	HER2 + cORR 59.4%; DCR 90.6%; PFS 8.6 mo (95% CI 5.4—NE) HER2 low cORR 37.5%; DCR 75%, PFS 6.3 mo (95% CI 2.3—NE) AE > = grade 3 in 43.8%. 5 patients with treatment-related ILD (one grade 5, 4 grade 2)
NCT04132960 DAISY	Mosele	2023	Phase II	Heavily pretreated	HER2+ , HER2 low or HER2 negative MBC		186	T-DXd	ORR HER2 + 70.6% (95% CI 58.3–81) ORR HER2 low 37.5% (26.4–49.7) ORR HER2- 29.7% (15.9–47)
NCT04420598 DEBBRAH cohort 5	Vaz Batista	2024	Phase II	Previously treated	HER2+ and HER2 low with leptomeningeal carcinomatosis		41	T-DXd	PFS 8.9 mo (95% CI 2.1-NE) OS 13.3 mo (2.5-NE) CBR 71.4%
NCT04752059 TUXEDO	Bartsch	2022	Phase II	Previously treated	HER2+ MBC with CNS metastasis		15	T-DXd	Intracranial ORR 73.3% (95% CI 48.1–89.1)
*Datopotamab deruxtecan (Dato-DXd)*									
NCT03401385 TROPION-PanTumour 01	Bardia	2024	Phase I	Heavily pretreated	a/m HR+ /HER2- or TNBC		85	Dato-DxD	ORR HR + /HER2: 26.8% (95% CI 14.2–42.9) PFS HR + /HER2- 8.3 mo ORR TNBC 31.8% (18.6–47.6) PFS TNBC 4.4 mo
NCT05104866 TROPION-Breast 01	Bardia	2023	Phase III	> First line	HR+ /HER2- MBC		732	Arm A: Dato-DXd Arm B: physician's choice of capecitabine, eribulin, vinorelbine, or gemcitabine	**PFS: 6.9 vs 4.9 mo, HR 0.63 (95% CI 0.52–0.76)** OS immature, HR 0.84 (0.62–1.14)
NCT03742102 BEGONIA Arm 6	Schmid	2023	Phase Ib/II	First line	HR-/HER2 low MBC		46	T-DXd + Durvalumab	ORR 57% (95% CI 41–71); mDOR NE; mPFS 12.6 mo (8-NE)
NCT03742102 BEGONIA Arm 7	Schmid	2023	Phase Ib/II	First line	a/m TNBC		62	Dato-DXd + Durvalumab	ORR 79% (95% CI 67–88); mDOR 15.5 mo (9.9—NC); mPFS 13.8 mo (11—NC)
NCT01042379 I-SPY2.2	Shatsky	2024	Phase II	Neoadjuvant	Stage II-III HER2- high-risk breast cancer		47	Dato-DXd + Durvalumab	overall pCR 50% pCR in immune phenotype 79% pCR in TNBC 62%
NCT05866432 TUXEDO-2	Bartsch	2024	Phase II	Previously treated	TNBC with CNS metastasis		8	Dato-DxD	intracranial response 37.5%
*Disatamab vedotin*									
NCT02881138 NCT03052634 C003 CANCER	Xu	2020	Phase Ib	Previously treated	HER2+ MBC		70	Disatamab vedotin	no DLT ORR 31.4% CBR 38.6% PFS 5.8 months
NCT05331326	Wu	2024	Phase II	Third line	HER2+ and HER2-low MBC with abnormal PAM pathway activation		62	Disatamab vedotin	ORR 34.4% PFS 3.5 mo (95% CI 2.4–4.6)
	Qu	2023	Phase II	Heavily pretreated	HER2+ or HER2 low MBC		120	Disatamab vedotin in combination with ICI, TKI, or chemotherapy	ORR 38.3% (95% CI 30.0–47.3) PFS 5.7 mo (4.6–6.9)
*Enfortumab vedotin*									
EV-202 NCT04225117	Giordano	2024	Phase II	Heavily pretreated	TNBC or HR+ /HER2- MBC		87	Enfortumab vedotin	TNBC ORR 19%, DCR 57.1%, PFS 3.5 mo (95% CI: 2.1–4.6), OS 12.9 mo (10.3-NE) HR + /HER2- ORR 15.6%, DCR 51.1%, PFS 5.4 mo (3.4–5.7), OS 19.8 mo (12.8-NE)
*Ladiratuzumab vedotin*									
NCT01969643 SGNLVA-001	Tsai	2021	Phase I	HR + /HER2-: second line TNBC: third line	LIV + HR + /HER2- and TNBC		81	Ladiratuzumab vedotin	no DLT ORR TNBC: 28% (95% CI 13–47) HR + /HER2- pending
NCT03310957 SGNLVA-002	Han	2020	Phase Ib/II	First line	mTNBC		51	Ladiratuzumab vedotin + pembrolizumab	ORR 54% (95% CI: 33.4–73.4)
NCT01042379 I-SPY2	Beckwith	2021	Phase II	Neoadjuvant	Stage II-III HER2- breast cancer		60	Ladiratuzumab vedotin followed by AC	predicted pCR overall: 0.16 (95% CI 0.08–0.24)
*Patritumab deruxtecan*									
NCT02980341	Krop	2023	Phase I/II	Heavily pretreated	MBC	HER3 +	182	Patritumab deruxtecan	HR + /HER2- ORR 30.1% (95% CI 21.8–39.4), DCR 80.5% (72.0–87.4), mPFS 7.4 mo TNBC ORR 22.6% (12.3–36.2), DCR 79.2% (65.9–89.2), mPFS 5.5 mo HER2 + ORR 42.9% (17.7–71.1), DCR 92.9% (66.1–99.8), mPFS 11.0 mo
ICARUS-BREAST-01 NCT04965766	Pistilli	2023	Phase II	> First line	HR + /HER2- MBC		56	Patritumab deruxtecan	3 mo RR 28.6% (95% CI 18.4–41.5), all partial response
SOLTI TOT-HER3 NCT04610528	Oliveira	2023	Phase I	Neoadjuvant/Window of Opportunity	HR + /HER2- or TNBC early breast cancer		37	single dose Patritumab deruxtecan 5.6 mg/kg	ORR 35% in TNBC, 30% in HR + /HER2- Change in cellularity and TIL associated with ORR (*p* = 0.049) No association between HER3 expression and ORR
*Sacituzumab tirumotecan*									
NCT05347134 OptiTROP-Breast01	Xu	2024	Phase III	Second line	a/m TNBC		263	Arm A: Sacituzumab tirumotecan Arm B: physician's choice of capecitabine, eribulin, gemcitabine or vinorelbine	PFS 5.7 (95% CI: 4.3–7.2) vs 2.3 mo (1.6–2.7) **OS NE (11.2-NE) vs 9.4 (8.5–11.7), HR 0.53 (0.36–0.78)**
*Trastuzumab duocarmazine (T-Duo)*									
NCT02277717	Banerji	2019	Phase I	Metastatic	HER2 + or HER2 low MBC		95	T-Duo	HER2 + ORR 33% (95% CI: 20.4–48.4) HR + /HER2 low ORR 28% (13.8–46.8) HR-/HER2 low ORR 40% (16.3–67.6)
NCT03262935 TULIP	Saura Manich Aftimos	2021 2023	Phase III	Previously treated	HER2 + MBC		437	Arm A: T-Duo Arm B: physician's choice of capecitabine + trastuzumab, eribulin + trastuzumab, vinorelbine + trastuzumab or capecitabine + lapatinib	**PFS 7.0 vs 4.9 mo (HR 0.63, p = 0.002)** OS 21.0 vs 19.5 mo, HR 0.87 (95% CI 0.68–1.12)
*Selected upcoming clinical trials*									
NCT05382299 ASCENT-03			Phase III	First line	mTNBC	PD-L1-	540	Arm A: SG Arm B: physician's choice (paclitaxel, nab-paclitaxel, or gemcitabine)	PFS
NCT05382286 ASCENT-04			Phase III	First Line	mTNBC	PD-L1 +	440	Arm A: SG + pembrolizumab Arm B: physician's choice (paclitaxel, nab-paclitaxel, or gemcitabine) + pembrolizumab	PFS
NCT05633654 ASCENT-05/Optimice-RD			Phase III	Adjuvant	Early TNBC with RCB		1514	Arm A: SG + Pembrolizumab Arm B: Pembrolizumab ± Capecitabine	iDFS
NCT06393374			Phase III	Adjuvant	Early TNBC with RCB		1530	Arm A: SG + Pembrolizumab Arm B: Pembrolizumab + Capecitabine	iDFS
NCT06393374			Phase II	Neoadjuvant	TNBC		260	Cohort 2: SG + pembrolizumab	pCR
NCT04468061 SACI-IO TNBC			Phase II	First Line	mTNBC	PD-L1-	110	Arm A: SG Arm B: SG + pembrolizumab	PFS
NCT03971409 InCITe			Phase II	First or second line	mTNBC		150	Arm A: binimetinib followed by binimetinib + avelumab + Liposomal Doxorubicin Arm B: SG followed by SG + avelumab Arm C: liposomal doxorubicin followed by liposomal doxorubicin + avelumab	ORR
NCT04595565 SASCIA			Phase III	Residual disease	HR + /HER2- or TNBC		1332	Arm A: SG Arm B: physician's choice (capecitabine, carboplatin or cisplatin ± pembrolizumab)	iDFS
NCT04448886			Phase II	First or second line	HR + /HER2- MBC		110	Arm A: SG + pembrolizumab Arm B: SG	PFS
NCT04647916			Phase II	Previously treated	HR + /HER2- or TNBC with CNS metastasis		44	SG	ORR
NCT06263543 SERIES			Phase II	Previously treated	ER + /HER2 low		75	SG	ORR
NCT05143229 ASSET			Phase I	Previously treated	a/m HER2- BC		18	SG + alpelisib	RP2D
NCT05675579			Phase II	Previously treated	early-stage TNBC		25	SG + pembrolizumab	Safety
NCT04039230			Phase I/II	Previously treated	mTNBC		75	SG + talazoparib	DLT
NCT04434040 ASPRIA			Phase II	Residual disease	TNBC		40	Atezolizumab + SG	rate of clearance of cfDNA
NCT03424005 MORPHEUS			Phase Ib/II	First line	a/m breast cancer Cohort 1: PD-L1 + TNBC Cohort 2: ICI-naïve TNBC Cohort 3: HR + /HER2-PIK3CA + Cohort 4: HER2 + or HER2low PIK3CA +		580	Cohort 1 Arm A: Atezolizumab + nab-paclitaxel Cohort 1 Arm B: Atezolizumab + Nab-Paclitaxel + Tocilizumab Cohort 1 Arm C: Atezolizumab + SG Cohort 2 Arm A: Capecitabine Cohort 2 Arm B: Atezolizumab + Ipatasertib Cohort 2 Arm C: Atezolizumab + LV Cohort 2 Arm D: Atezolizumab + Selicrelumab + Bevacizumab Cohort 2 Arm E: Atezolizumab + Chemo (Gemcitabine + Carboplatin or Eribulin) Cohort 3 Arm A: Inavolisib + Abemaciclib + Fulvestrant Cohort 3 Arm B: Inavolisib + Ribociclib + Fulvestrant Cohort 4 Arm A: Inavolisib + Trastuzumab Deruxtecan Cohort 4 Arm B: Inavolisib + Ribociclib + Letrozole Cohort 4 Arm C: Inavolisib + Ribociclib + Fulvestrant Cohort 4 Arm D: Inavolisib + Abemaciclib + Letrozole Cohort 4 Arm E: Inavolisib + Trastuzumab Deruxtecan	ORR
NCT04873362 ASTEFANIA			Phase III	Adjuvant	Early Breast Cancer with RCB	Any	1700	Arm A: T-DM1 + Atezolizumab Arm B: T-DM1 + Placebo	iDFS
NCT04740918 KATE3			Phase III	First to third line	Advanced/Metastatic	PD-L1 +	350	Arm A: T-DM1 + Atezolizumab Arm B: T-DM1 + Placebo	PFS and OS
NCT04622319 DESTINY-Breast 05			Phase III	First line	Early HER2 + Breast cancer with RCB		1600	Arm A: T-DXd Arm B: T-DM1	iDFS
NCT04538742 DESTINY-Breast 07			Phase Ib/II	Part 1: Previously Treated Part 2: First line	HER2 + MBC		245	Arm A: T-DXd Arm B: T-DXD + durvalumab Arm C: T-DXd + pertuzumab Arm D: T-DXd + paclitaxel Arm E: T-DXd + durvalumab + paclitaxel Arm F T-DXd + tucatinib	Safety
NCT04556773 DESTINY-Breast 08			Phase I	Part 1: Previously treated Part 2: First line	HER2 low MBC		138	Arm A: T-DXd + capecitabine Arm B: T-DXd + durvalumab + paclitaxel Arm C: T-DXd + capivasertib Arm D: T-DXd + anastrazole Arm E: T-DXd + fulvestrant	Safety
NCT04784715 DESTINY-Breast 09			Phase III	First line	HER2 + MBC		1157	Arm A: T-DXd + placebo Arm B: T-DXd + pertuzumab Arm C: taxane + pertuzumab + trastuzumab	PFS
NCT05113251 DESTINY-Breast 11			Phase III	First line	Neoadjuvant HER2 +		927	Arm A: T-DXd Arm B: T-DXd followed by taxane + pertuzumab + trastuzumab Arm C: doxorubicin + cyclophosphamide followed by taxane + pertuzumab + trastuzumab	pCR
NCT04739761 DESTINY-Breast 12			Phase III	> First line	HER2 + MBC ± CNS metastasis		506	T-DXd	ORR and PFS
NCT05950945 DESTINY-Breast 15			Phase III	> First line	HER2 low or HER2- MBC		250	T-DXd	Time to Initiation of Subsequent Anticancer Treatement
TRIO-US B-12/TALENT NCT04553770			Phase II	Neoadjuvant	HR + /HER2 low		88	Arm A: T-DXd Arm B: T-DXd + anastrazole	pCR
TRANSCENDER NCT05744375			Phase II	First line	a/m HER2 + with early relapse < 12 mo		41	T-DXd	ORR
NCT03742102 BEGONIA			Phase I/II	First line	mTNBC		243	Arm 1: durvalumab + paclitaxel Arm 2: capivasertib + durvalumab + paclitaxel Arm 5: durvalumab + oleclumab + paclitaxel Arm 6: durvalumab + T-DXd Arm 7: durvalumab + Dato-DXd Arm 8: durvalumab + Datopotomab deruxtecan in PD-L1 +	Safety
NCT05374512 TROPION-Breast 02			Phase III	First line	mTNBC	PD-L1-	637	Arm A: Dato-DXd Arm B: physician's choice of paclitaxel, nab-paclitaxel, capecitabine, carboplatin or eribulin	PFS OS
NCT05629585 TROPION-Breast 03			Phase III	Residual disease	adjuvant TNBC		1075	Arm A: Dato-DXd + Durvalumab Arm B: Dato-DXd Arm C: physician's choice of capecitabine and/or pembrolizumab	iDFS
NCT06112379 TROPION-Breast-04			Phase III	Neoadjuvant + Adjuvant	Stage II-III TNBC or HR-low, HER2 negative		1728	Arm A: Dato-DXd + Durvalumab → Adjuvant Durvalumab ± Chemotherapy Arm B: Carboplatin + Paclitaxel + 4xAC/EC + Pembrolizumab → adjuvant Pembrolizumab	pCR and EFS
NCT06103864 TROPION-Breast 05			Phase III	First Line	a/mTNBC	PD-L1 +	635	Arm A: Dato-DXd + Durvalumab Arm B: physician's choice of chemotherapy (paclitaxel, nab-paclitaxel, gemcitabine or carboplatin) with pembrolizumab	PFS
NCT06176261 DATO-BASE			Phase III	ER + /HER2-: Prior endocrine therapy TNBC or HER2-: Any	HER2- MBC with CNS metastasis		58	Dato-DXd	ORR
NCT05460273 TROPION-PanTumour02 Cohort 2			Phase I/II	Third Line	a/mTNBC		78	Dato-DXd	ORR
NCT06508216 COMPASS-TNBC			Phase I/II	First Line	mTNBC with early relapse < 12 mo		60	Arm A: Dato-DXd Arm B: Dato-DXd + Durvalumab	ORR
NCT06533826 TRADE-DXd			Phase II	First to third line	HER2 low a/MBC		357	Arm A: T-DXd. After progression, then Dato-DXd Arm B: Dato-DXd. After progression, then T-DXd	ORR
NCT06157892			Phase Ib/II	Second or third line	HER2 + or HER2 low MBC		198	Arm A: Disatamab vedotin Arm B: Disatamab vedotin + tucatinib	DLT ORR
NCT05726175			Phase II	Neoadjuvant	stage II-III HER2 low breast cancer		20	Arm A: Disatamab vedotin + penpulimab	pCR
NCT06178159			Phase II	Neoadjuvant	HER2 + breast cancer		80	Arm A: Disatamab vedotin + pertuzumab Arm B: Disatamab vedotin + pertuzumab + toripalimab	pCR
NCT06227117			Phase II	Neoadjuvant	stage II-III HR-/HER2 + breast cancer		120	Arm A: Disatamab vedotin + toripalimab Arm B: carboplatin + disatamab vedotin + toripalimab Arm C: Disitamab Vedotin + toripalimab then EC + toripalimab	pCR
NCT06000033			Phase II	Third line	HR-/HER2 low MBC		35	Disitamab vedotin + anlotinib	ORR
NCT05831878			Phase II	Second line	HR-/HER2 low MBC		36	Disatamab vedotin	ORR
NCT03500380			Phase II/III	Previously treated	HER2 + MBC ± liver metastasis		301	Arm A: Disatamab vedotin Arm B: capecitabine + lapatinib	PFS
NCT06157892 Rosy			Phase III	Previously treated	HR + /HER2 low MBC		288	Arm A: Disatamab vedotin Arm B: physician's choice of endocrine therapy	PFS
NCT04400695			Phase III	Second line	HER2 low MBC		366	Arm A: Disatamab vedotin Arm B:physician's choice of capecitabine, docetaxel, paclitaxel, or vinorelbine	PFS
NCT04300556			Phase I/II	> First line	Solid tumors including mTNBC		142	Farletuzumab ecteribulin	ORR DLT Safety
NCT05865990			Phase II	TNBC: > 1st line HER2+ : > 2nd line	MBC and NSCLC with CNS metastasis		60	Patritumab deruxtecan	Intracranial ORR OS
NCT04699630			Phase II	TNBC: 2nd line HER2+ : > 2nd line including T-DXd	MBC		121	Patritumab deruxtecan	ORR PFS
NCT06298084 ICARUS-BREAST-02			Phase Ib/II	Previously treated, including with T-DXd	MBC		152	Patritumab deruxtecan + olaparib	Safety, ORR, DOR, PFS, CBR
NCT05569811 VALENTINE			Phase II	Neoadjuvant	HR + /HER2-; Ki67 > 20% and/or high genomic risk		120	Arm A: chemotherapy Arm B: Patritumab deruxtecan + letrozole Arm C: Patritumab deruxtecan	pCR
NCT06312176 TroFuse-010			Phase III	Previously treated	HR + /HER2- MBC		1200	Arm A: Sacituzumab tirumotecan Arm B: Sacituzumab tirumotecan + pembrolizumab Arm C: physician's choice of capecitabine, liposomal doxorubicin, paclitaxel, or nab-paclitaxel	PFS
NCT06393374			Phase III	Adjuvant	Early TNBC with RCB		1530	Arm A: Sacituzumab tirumotecan + pembrolizumab Arm B: physician's choice of capecitabine or capecitabine + pembrolizumab	iDFS
NCT06312176			Phase III	Previously treated	Advanced or Metastatic	1200	1200	Arm A: Sacituzumab Tirumotecan Arm B: Sacituzumab Tirumotecan + Pembrolizumab Arm C: Treated of Physician's Choice	PFS
NCT01042379 I-SPY2			Phase II	Neoadjuvant	Stage II-III HER2- breast cancer			T-Duo	predicted pCR

Results in bold are statistically significant

### HER2+ vaccines

Several HER2-directed short peptide vaccines have been developed, with E75 being the most extensively studied. An optimal regimen of NP-S with GM-CSF monthly for 6 months was determined in a phase I/II study [131], with a trend towards increased 5-year DFS in the vaccinated group vs. control. In the phase III setting, however, no difference in 3-year DFS was seen and the trial was stopped early for futility [132]. HER2 vaccines targeting AE37 have not shown any difference in DFS [133]. GLSI-100, a HER2 vaccine targeting GP2, has been investigated in the adjuvant setting for patients with HER2+ , node-positive or otherwise high-risk disease [133]. Overall, there was no difference in 5-year DFS, but patients who had disease that was HER2 3+ by IHC did have a trend toward significant improvement in DFS [133]. This vaccine is being investigated in the upcoming phase III Flamingo-01 (NCT0523916) study in the adjuvant setting for patients with HER2+ residual disease (Table 6).

**Table 6:** Current and upcoming clinical trials of vaccines in breast cancer

Trial name	Primary author	Year	Study design	Line of Therapy	Setting	TAAs/MOA	# Patients	Drug regimen	Results
*Peptide vaccines*									
NCT00003638 Theratrope	Miles	2011	Phase III	> First Line	Metastatic	Muc-1	1028	Arm A: sialyl-TN conjugated to keyhole limpet hemocyanin (KLH) protein + cyclophosphamide Arm B: Placebo KLH protein + cyclophosphamide	6 mo PFS: 53% vs 33% (*p* = 0.011) TTP 3.4 vs 3.0 mo (p = 0.353) OS 23.1 mo vs. 22.3 (*p* = 0.916)
NCT01516307	Huang	2020	Phase II	First or Second line	Metastatic with stable/responding disease	Ada-Sim / OBI-821	348	Arm A: adagloxad simolenin + cyclophosphamide Arm B: placebo + cyclophosphamide	PFS: 7.6 mo vs 9.2 mo (HR 0.96, 95% CI 0.74–1.25) **Anti-Globo-H IgG titer > = 1:160 vs < 1:160: PFS 11.1 mo (9.3–17.6) vs 5.5 mo (3.7–5.6), HR 0.52, *p* < 0.0001)**
NCT01532960	Dillon	2017	Phase I	First line	Adjuvant	MAGE CEA NY-ESO-1 HER2	12	Poly-ICLC vaccine	Response Rate 0% Terminated for futility
NCT02364492	Rosenbaum	2020	Phase I	First line	Adjuvant, HER2 negative at high risk for relapse	MAGE	7	MAG-Tn3/AS15 vaccine	all vaccinated patients developed high levels of Tn-specifc antibodies
NCT01220128	Higgins	2017	Phase II	Neoadjuvant	Stage II or III breast cancer expressing WT1	WT1	66	Arm A: Standard neoadjuvant therapy + WT1 ASCI Arm B: Standard neoadjuvant therapy + Placebo	Terminated
NCT02229084	Makhoul	2021	Phase I/II	Neoadjuvant	Stage I-III ER + /HER2- breast cancer	Peptide Vaccine	25	Standard Neoadjuvant Therapy + P10s-PADRE	Increase in CD16, NKp46 and CD94 expression on NK cells, increased IFN-γ
*HER2 vaccines*									
NCT00841399 NCT00584789	Mittendorf	2014	Phase I/II	First line	Adjuvant, HER2 IHC 1–3 +	E75/NeuVax	195	Arm A: NP-S + GM-CSF Arm B: Control	5-year DFS: 89.7% vs 80.2% (*p* = 0.08)
NCT01479244 PRESENT	Mittendorf	2019	Phase III	First line	Adjuvant, T1-3, N1-3, HER2 low (IHC 1 + /2 +)	E75/NeuVax	758	Arm A: NP-S + GM-CSF Arm B: placebo + GM-CSF	Recurrence Rate at 16.8 months: 9.8% vs 6.3% (*p* = 0.07) 3-year DFS 77.1% vs 77.5% Stopped early for futility
NCT00524277	Brown	2020	Phase II	First line	Adjuvant, HER2 IHC 1–3 + node-positive or high-risk node negative	GP2	180	GP2 + GM-CSF	5-year DFS 82.9% (95% CI 75–91) vs 80.4% (69–88), HR .967 (0.460–2.034) HER2 (IHC 3 +) subgroup: DFS 100% vs 87.2% (71–95), *p* = 0.052)
NCT00524277	Brown	2020	Phase II	First line	Adjuvant, HER2 IHC 1–3 + node-positive of high-risk node negative	AE37	298	AE37 + GM-CSF	recurrence rate 12.4% vs 13.8%, HR 0.885 (95% CI 0.472–1.659) 5-year DFS 80.1% vs 79.3%, HR 0.989 (0.588–1.665)
*Viral Vaccines*									
	Mohebtash	2011	Phase I	Heavily pretreated	Metastatic	Muc-1 CEA co-stimulatory molecules	12	PANVAC monthly	median time to progression 2.5 mo OS 13.7 mo One patient with complete response Four patients with stable disease
NCT00179309	Heery	2015	Phase II	Any	Metastatic	Muc-1 CEA co-stimulatory molecules	48	Arm A: Docetaxel + PANVAC Arm B: Docetaxel	PFS 7.9 mo vs 3.9 mo (HR 0.65 (95% CI 0.34–1.14)
NCT01656538 IND-213	Bernstein	2018	Phase II	Any	Metastatic	Type 3 Reovirus	72	Arm A: Paclitaxel Arm B: Paclitaxel + Pelareorep	PFS 3.78 mo vs 3.38 mo, HR 1.04 (80% CI 0.76–1.43) OS 17.4 mo vs 10.4 mo, HR 0.65 (0.46–0.91)
NCT03387085	Nangia	2019	Phase Ib	Third line	Metastatic TNBC	Adenovirus CEA, MUC1, brachyury and HER2; yeast vector Ras, brachyury and CEA	8	chemotherapy + bevacizumab + SBRT + vaccines + avelumab	No dose limiting toxicities One complete response, and 2 partial responses
NCT04102618 AWARE-1/REO-027	Manso	2020	Phase I	Neoadjuvant	Early- Stage TNBC Window of Opportunity	Type 3 Reovirus	38	Pelareorep + Atezolizumab	Increase in caspase 3 staining (*p* = 0.04) Decrease in T cell diversity (*p* = 0.01)
NCT03674827	Pfizer	2022	Phase I	Third line	Metastatic TNBC or NSCLC	Viral Vaccine	36	VBIR-2 + Tremelimumab + Sasanlimab	No DLT
NCT04215146 Bracelet-1	Clark	2023	Phase II	Any	Metastatic HR+ /HER2-	Type 3 Reovirus	48	Arm A: Paclitaxel Arm B: Paclitaxel + Pelareorep Arm C: Paclitaxel + Pelareorep + Avelumab	ORR: 20% vs 31.3% vs 17.6% PFS: 6.4 mo vs 9.6 mo vs 7.5 mo OS pending
NCT02779855	Soliman	2023	Phase II	Neoadjuvant	Stage II-III TNBC	oncolytic herpesvirus	37	Intratumoral talimogene laherparepvec (T-VEC) + Paclitaxel followed by AC	pCR 45.9% (90% CI 32–54) 2-year DFS 89%
NCT03256344	Hecht	2023	Phase Ib	Any	Metastatic TNBC with liver metastasis	oncotypic herpesvirus	11	Intratumoral T-VEC + Atezolizumab	No DLT ORR 10% (95% CI 0.3–44.5) One patient with PR
NCT03567720 KEYNOTE-890 Cohort 1	Telli	2021	Phase II	Second line	Advanced/Metastatic TNBC	Plasmid	26	Tavokinogene telseplasmid (TAVO-EP) + Pembrolizumab	ORR 17.4% One patient with CR, 4 patients with PR OS 11.0 mo
*Dendritic Cell Vaccine*									
NCT001070211	Sharma	2012	Pilot	First line	HER2 + DCIS	HER2/neu	27	HER2/neu DC vaccine	5/27 patients with no residual disease at time of surgery
NCT02061332	Lowenfeld	2017	Phase I/II	First line	HER2 + DCIS or early breast cancer	HER2/neu	54	Arm A: Intralesional administration HER2/neu DC vaccine Arm B: Intranodal administration HER2/neu DC vaccine Arm C: Intralesional and intranodal administration HER2/neu DC vaccine	IRR 84.2% (95% CI 60.4–96.6) vs 89.5% (66.9–98.7) vs 66.7% (38.4–88.2) pCR 28.6% in patients with DCIS pCR 8.3% for patients with breast cancer
	Svane	2007	Phase II	Heavily pretreated	HLA-A2 + advanced breast cancer	p53	26	p53-peptide loaded DC vaccine	8/19 stable disease
NCT01042535	Soliman	2018	Phase I/II	Pretreated	ER + /HER2- Advanced breast cancer	p53	9	p53-peptide loaded DC vaccine + indoximod	best respose stable disease
NCT02018458	O'Shaughnessy	2016	Phase I/II	Neoadjuvant	Locally advanced TNBC	Cyclin B1, WT1 26, CEF	10	ddAC followed by paclitaxel and carboplatin + DC vaccine	pCR 50%
*Selected Upcoming Vaccine Clinical Trials*									
NCT0523916 FLAMINGO-01			Phase III	Adjuvant	Early Breast Cancer with RCB	HER2 Vaccine	498	Arm A: GLSI-100 Arm B: Placebo	iBCFS
NCT03562637 GLORIA			Phase III	Adjuvant	TNBC expressing GloboH at high risk for recurrence	Ada-Sim / OBI-821 vaccine	668	Arm A: adagloxad simolenin + standard of care Arm B: standard of care alone	iDFS
NCT03567720 KEYNOTE-890 Cohort 2			Phase II	First line	Metastatic TNBC	Intratumoral IL-12	40	TAVO-EP + Pembrolizumab + nab-paclitaxel	ORR
NCT04348747			Phase II	Previously treated	Metastatic TNBC or HER2 + with untreated brain metastasis	HER2/3 Dendritic Cell Vaccine	21	alphaDC-1 + CKM + Pembrolizumab	RR
NCT03606967			Phase II	First line	mTNBC	Peptide Vaccine	70	Arm A: nab-paclitaxel + durvalumab + neoantigen vaccine Arm B: nab-paclitaxel + durvalumab	PFS
NCT03362060			Phase Ib	> First line	mTNBC HLA-A2 +	Peptide Vaccine	20	PVX-410 Vaccine + Pembrolizumab	PFS
NCT02826434			Phase Ib	Prior neoadjuvant or adjuvant therapy	Stage II/III TNBC, HLA-A2 +	Peptide Vaccine	22	PVX-410 Vaccine + Durvalumab	DLT
NCT05269381			Phase I	> First line	Advanced TNBC or other solid tumors	Peptide Vaccine	36	cyclophosphamide followed by vaccine + GM-CSF + Pembrolizumab	Safety
NCT04024800			Phase II	First line	mTNBC	HER2 Vaccine	29	AE37 + Pembrolizumab	ORR
NCT05455658			Phase II	Adjuvant	Stage IB-III TNBC	Plasmid Vaccine	33	STEMVAC (sargramostim)	Cellular Immune Response
NCT06435351			Phase I	Adjuvant	Early TNBC with RCB	Dendritic Cell Vaccine	16	DC vaccine based on whole exon sequencing of tumor with pembrolizumab and/or capecitabine	Feasibility

Results in bold are statistically significant

### Upcoming clinical trials in HR-/HER2+ breast cancer

The ASTEFANIA (NCT04873362) phase III trial is investigating T-DM1 with or without atezolizumab in the adjuvant setting for patients with residual disease after neoadjuvant therapy, with the goal to enhance the current standard of care for patients with RCB in the adjuvant setting. KATE3 (NCT04740918) is a phase III trial evaluating the KATE2 regimen in patients with PD-L1+ disease in the first to third line a/m setting. Another phase III trial (NCT03199885) is investigating paclitaxel with trastuzumab, pertuzumab, and atezolizumab or placebo in the first line metastatic setting, with the goal of establishing a role for ICIs in a/m HER2+ breast cancer.

## Bispecific antibodies and cellular therapies

Immunotherapies other than immune checkpoint inhibitors seek to induce a durable immunologic anti-cancer response. Areas of research in breast cancer include bispecific antibodies as well as cellular therapies including tumor infiltrating lymphocytes (TILs), chimeric antigen receptor T (CAR-T), and T cell receptor engineered (TCR) treatments.

### Bispecific antibodies

Bispecific antibodies are engineered antibodies that simultaneously bind to two targets, typically a tumor-specific antigen on malignant cells and an immune cell [134]. Bringing these targets in close proximity engages the immune system, inciting apoptosis, enhanced immune activation signaling, or reducing immunosuppressive factors, all of which promote anti-tumor activity [134]. The first FDA approval for a bispecific antibody in oncology was blinatumomab, which targets CD3 and CD19, in B-cell acute lymphoblastic leukemia in 2017 [135]. Bispecifics have been an area of active research in breast cancer for over 30 years. TAAs targeted by bispecific therapies in breast cancer research include HER2, HER3, PD-L1, and CD3 [134].

HER2 is the most commonly studied bispecific TAA in breast cancer, in combination with another HER2 antibody, HER3, CD3, and CD16. Zanidatamab, a dual-HER2 bispecific antibody, is efficacious in both advanced and early-stage HER2+ BC. A phase I clinical trial of zanidatamab in advanced HER2+ solid cancers, including breast cancer, found an ORR of 37% with zanidatamab monotherapy [136]. Zanidatamab in combination with docetaxel had an 90.9% ORR in patients with advanced HER2+ mBC [137]. In HR+ /HER2+ patients with advanced disease, the combination of zanidatamab with palbociclib and fulvestrant achieved a median PFS of 11.3 months with an ORR of 34.5% [138]. In the neoadjuvant setting, patients with stage 1, node-negative HER2 + BC were treated with zanidatamab in a chemotherapy-free regimen, with preliminary data showing 36% of patients achieving pCR and 64% of patient achieving pCR or RCB-1 [139]. An upcoming phase III trial (NCT06435429) will investigate chemotherapy with zanidatamab or trastuzumab for patients with HER2+ disease who progress on T-DXd.

KN026, another dual-HER2 bispecific antibody, has found success in a phase I trial of patients with HER2+ mBC. Overall, a 28.1% ORR and median PFS of 6.8 months was found, though patients with HER2+ and CDK12 co-amplification had a significantly improved response (ORR of 50%, median PFS 8.2 months) [140]. This improved response is thought to be because both HER2 and CDK12 genes are located on chromosome 17, approximately 200 kb apart. KN026 in combination with KN046, an anti-PD-L1/CTLA-4 bispecific antibody, found an ORR of 50% in patients with HER2 + MBC [141]. In the neoadjuvant setting, KN026 with docetaxel achieved a pCR rate of 56.7% [142]. A trial of KN026 with palbociclib and fulvestrant (NCT04778982) was terminated due to low enrollment. An upcoming phase I clinical trial (NCT03842085) will investigate another dual HER2 bispecific antibody, MBS301, in HER2+ solid tumors.

Bispecific antibodies targeting HER2 and HER3 include MCLA-128/Zenocutuzumab and MM-111. A phase II trial of zenocutuzumab with endocrine therapy in patients with HR+ , HER2-low MBC previously treated with CDK4/6 therapy found an DCR of 45% [143], while zenocutuzumab with trastuzumab and vinorelbine in HER2+ MBC previously treated with T-DM1 found an DCR of 77% [144]. MM-111 has also proven to be safe in phase I clinical trials [145, 146], though without further investigation in phase II or beyond. HER2 and CD3 bispecific antibodies including ertumaxomab [147], p95HER2 [148], and GBR1302 [149] have been investigated, but are not moving forward in current clinical trials.

Bispecific antibody armed T cells targeting HER2 and CD3, referred to as HER2 BATs, were investigated in a phase II clinical trial of treatment consolidation with immunotherapy after chemotherapy in HER2- MBC [150]. Evidence of immune activation in both the adaptive and innate response were seen, with a median OS of 13.1 months [150]. The role of HER2 BATs in HER2+ disease was evaluated in a phase I clinical trial, which showed that patients with higher HER2+ expression (IHC 3+) had a longer OS in comparison to patients with HER2 0 to 2+ expression (57 months vs. 27 months) [151]. The combination of HER2 BATs with pembrolizumab will be investigated in an upcoming phase I/II study (NCT03272334) (Table 7).

**Table 7:** Current and upcoming clinical trials of bispecific antibodies in breast cancer

Trial name	Primary author	Year	Study design	Line of therapy	Setting	Mechanism of Action: immune-cell engager (ICE), T-cell engager (TCE), NK engager, dual tumor associated antigen (TAA)	TAA	# Patients	Drug regimen	Results
*Bispecific antibody*										
NCT05035836	Valero	2023	Phase II	Neoadjuvant	Stage I HER2 + breast cancer	dual TAA	HER2 x HER2	11	Zanidatamab	pCR 36%; pCR/RCB1 64%
NCT04224272	Escrivá-de-Romani	2023	Phase II	Previously treated	Advanced HR + /HER2 + breast cancer	dual TAA	HER2 x HER2	34	Zanidatamab + Fulvestrant + Palbociclib	DLT in one patient (neutropenia) cORR 34.5% (95% CI 17.9—54.3) mPFS 11.3 mo (CI 5.6—NE)
NCT02892123	Meric-Bernstam	2022	Phase I	Heavily pretreated	Advanced/Metastatic HER2 + solid tumors, including MBC	dual TAA	HER2 x HER2	279	Zanidatamab + chemotherapy	DLT not reached; grade 3 + AE 3% of patients ORR 37% (95% CI 27.0–48.7) Part 3 ongoing
NCT04276493	Wang	2023	Phase Ib/II	Previously treated	Advanced HER2 + breast cancer	dual TAA	HER2 x HER2	37	Zanidatamab + Docetaxel	ORR = 90.9% (95% CI 75.7–98.1) 67.6% with grade 3 + treatment-related AE
NCT03619681	Zhang	2022	Phase I	Heavily pretreated	HER2 + MBC	dual TAA	HER2 x HER2	63	KN026	ORR 28.1% PFS 6.8 mo (95% CI 4.2–8.3) CDK12 amplified subgroup ORR 50% vs 0% (*p* = 0.05), PFS 8.2 mo vs 2.7 mo (p = 0.04)
NCT04881929	Ma	2023	Phase II	Neoadjuvant	Stage II-III HER2 + breast cancer	dual TAA	HER2 x HER2	30	KN026 + Docetaxel	pCR 56.7% (95% CI 37.43–74.53) ORR 90% (73.47–97.89) Grade 3 + AE 53.5%
NCT04521179	Liu	2022	Phase II	Previously treated	HER2 + breast cancer	dual TAA	HER2 x HER2	36	KN026 + KN046	ORR 50% (95% CI 28.2–71.8) DCR 81.8% (59.7–94.8) PFS 5.6 mo (2.5—NE)
NCT02912949	Schram	2022	Phase II	Heavily pretreated	HER2 + breast cancer expressing NRG1 mutation	dual TAA	HER2 x HER3	5	Zenocutuzumab	Response in 2/4 evaluable patients
NCT03321981	Pistilli	2020	Phase II	Previously treated with CDK4/6i	HR + , HER2-low mBC	dual TAA	HER2 x HER3	48	Zenocutuzumab + Endocrine Therapy	DCR 45% (90% CI 32–59); 2 patients with partial response
NCT03321981	Hamilton	2020	Phase II	Previously treated with anti-HER2 ADC	HER2 + MBC	dual TAA	HER2 x HER3	28	Zenocutuzumab + Vinorelbine + Trastuzumab	DCR 77% (90% CI 60–89); 1 patient with complete response and 4 patients with partial response
NCT01304784	Richards	2014	Phase I	Previously treated	HER2 + MBC	dual TAA	HER2 x HER3	46	Arm A: MM-111 + Cisplatin + Capecitabine + Trastuzumab Arm B: MM-111 + Lapatinib ± Trastuzumab Arm C: MM-111 + Paclitaxel + Trastuzumab Arm D: MM-111 + Lapatinib + Trastuzumab + Paclitaxel Arm E: MM-111 + Docetaxel + Trastuzumab	MTD not met
NCT00911898	Beeram	2010	Phase I	Heavily pretreated	HER2 + advanced breast cancer	dual TAA	HER2 x HER3	11	MM-111	No DLT
NCT01097460	Higgins	2011	Phase I/II	Heavily pretreated	HER2 + MBC	dual TAA	HER2 x HER3	16	MM-111 + Herceptin	No results available
*NCT01569412*	Haense	2016	Phase I	Heavily pretreated	HER2 + MBC	TCE	HER2 x CD3	5	Ertumaxomab	One partial response No dose-limiting toxicities
NCT02829372	Wermke	2018	Phase I	Heavily pretreated	HER2 Positive solid tumor	TCE	HER2 x CD3	19	GBR 1302	Grade 1–2 CRS common; two patients with DLT. One patient with breast cancer has ongoing response at 4 mo
NCT00027807	Lum	2014	Phase I	Heavily pretreated	MBC	TCE	BATs (HER2 x CD3)	23	HER2Bi + IL-2 + G-CSF	Best response: Stable disease or better: 54.5% HER2 3 + OS 57.4 mo HER2 0–2 + OS 27.4 mo
NCT01022138	Lum	2021	Phase II	Heavily pretreated	Metastatic HER2/HR+ and TNBC with stable disease	TCE	BATs (HER2 x CD3)	32	HER2Bi + IL-2 + G-CSF	Overall OS 13.1 mo (95% CI 8.6–17.4) HER2-/HR + OS 15.2 mo (CI 8.6–19.8) TNBC OS 12.3 mo (2.1–17.8) Significant increases in interferon-γ immunospots, Th1 cytokines, Th2 cytokines, and chemokines after treatment
NCT02659631	Harding	2022	Phase I	Heavily pretreated	Advanced Solid Tumors	TCE	CD3 x p-cadherin	5	PF-06671008	Best response: stable disease
*Selected upcoming clinical trials*										
NCT06435429			Phase III	Pretreated	HER2 + MBC	dual TAA	HER2 Bispecific	550	Arm A: Chemotherapy + Zanidatamab Arm B: Chemotherapy + Trastuzumab	PFS
NCT05027139			Phase I/II	Pretreated	HER2 + solid tumors, including MBC	dual TAA	HER2 Bispecific + Anti-CD47	52	Zanidatamab + Evorpacept (ALX148)	Safety ORR
NCT03842085			Phase I	Metastatic	HER2 + solid tumors, including HER2 + MBC and HER2 low MBC	dual TAA	HER2x HER2	34	MS301	DLT
NCT03272334			Phase I/II	Second line	MBC	dual TAA	HER2 Bispecific	33	HER2Bi + Pembrolizumab	MTD
NCT04143711			Phase I/II	Previously treated	Advanced HER2 + solid tumors, including breast	NK cell engager	trispecific NK cell engager	378	Arm A: DF1001-001 Arm B: DF1001-001 + nivolumab Arm C: DF1001-001 + nab paclitaxel Arm D: DF1001-001 + sacituzumab govitecan	DLT Safety ORR

### Adoptive cell therapy: TILS, CAR-T, and TCR

While ICIs have made tremendous impact on outcomes in breast cancer, a significant subset of patients do not benefit from ICI. This is likely due, in part, to immune escape through defects in antigen presentation and other abnormal signaling pathways [152]. Mechanisms to overcome this barrier include adoptive cell transfer, which consists of infusing antigen-specific T cells into the patient, inducing an amplified immune response. Therapies such as TILs, CAR-T, and TCR cell therapies involve harvesting T cells from the patient, allowing for ex vivo expansion with or without genetic modification, and infusing these T cells back into the patient. Since the first FDA approval of CD-19-specific CAR-T therapy for B-cell acute lymphoblastic leukemia in 2017 [153], cellular therapy has changed the treatment paradigm in hematologic malignancies.

TIL therapy involves extracting T cells from within a tumor, multiplying them without genetic modification, then infusing them back into the patient. TIL therapy gained FDA approval for relapsed/refractory metastatic melanoma in February 2024 based on an ORR of 31.5% including three complete responses in seventy-three patients treated with lifeleucel, an autologous TIL therapy, in a phase II study (NCT02360579) [154, 155], with four-year follow up confirming a median OS of 13.9 months and 20.8% of patients having an ongoing response at 48 months [156]. This success has prompted investigation of TIL therapy for other solid malignancies, including breast cancer. A phase I trial of TIL therapy with IL-2 and pembrolizumab in breast cancer showed promising results, with three out of six patients having a response including 1 complete response [157]. Upcoming trials of TIL therapy in breast cancer include a phase II trial (NCT04111510) is investigating LN-145 TIL therapy in mTNBC, a phase I/II (NCT05451784) trial of PD-L1+ TILs in TNBC, a phase Ib (NCT05576077) study of TBio4101 with pembrolizumab in solid tumors including breast cancer, and a phase II (NCT03449108) trial of LN-145-S1 with ICI in metastatic TNBC.

CAR-T cell therapy engineers T-cell receptors to target a tumor-specific antigen on the cell surface. CAR-T is a staple in the treatment of hematologic malignancies, and is an area of active research in solid tumors including breast cancer. Phase I studies to date have demonstrated tolerable safety [158–160] with some patients experiencing stable disease [161]. A phase 0 trial of intratumorally injected CAR T cells against c-MET, a cell-surface molecule, in patients with mBC showed evidence of an inflammatory response within tumors, but no clinical response [158]. These results led to a phase I study of intravenous CAR-T targeting cMET, with two of four patients with TNBC experiencing stable disease at day 25 [161]. Upcoming studies of CAR-T in breast cancer include a phase I/II (NCT04020575) study targeting metastatic breast cancer expressing Muc1 growth factor receptor, called Muc1*, and a phase I study (NCT02706392) of CAR-T in liquid and solid malignancies expressing ROR1.

It is postulated that the limited efficacy of CAR-T in solid tumors to date may relate to tumor antigen modulation, insufficient specificity of target antigens, T cell exhaustion, poor cell trafficking to the tumor site, and dysregulation of effector function by the TIME [162–164]. Limited expansion of CAR-T cells after transfer has limited efficacy while also limited toxicity. Thus, concern remains for higher on-target off-tumor toxicity with greater CAR-T cell expansion, as seen in a patient treated with ERBB2-CAR-T therapy who experienced fatal cytokine release syndrome from on-target, off-tumor effect [165]. In response, it is now understood that CAR-T therapy targets should be restricted to cell-surface antigens with limited off-tumor expression, of which there are few [166]. Other toxicities from CAR-T therapy such as macrophage activation syndrome and immune cell-associated neurotoxicity syndrome also affect normal, healthy tissue.

TCR is similar to CAR-T, but expresses human leukocyte antigen (HLA)-restricted T-cell receptors, which allow for tumor-specific binding to both membrane and intracellular proteins expressed by the major histocompatibility complex (MHC), and thus limits toxicity to other healthy cells. The SPEARHEAD-1 (NCT03132922) phase II trial of afamitresgene autoleucel (“afami-cel”), an HLA-A*02 restricted TCR targeting MAGE-4 in patients with synovial sarcoma or myxoid round cell liposarcoma, found an ORR of 37% and a duration of response of 28.1 months [167]. Afami-cel received FDA approval for MAGE-4 expressing sarcoma in August 2024. Ongoing phase I trials of MAGE-A1 and MAGE-A3 have found tolerable safety and potential clinical efficacy in various solid tumors, without reported results for patients with breast cancer [168, 169]. Another TCR therapy, letetresgene autoleucel (“lete-cel”) targeting HLA-A*02 restricted NY-ESO1, found an ORR of 40% in the IGNYTE-ESO (NCT03967223) phase II trial of patients with synovial sarcoma and myxoid round cell liposarcoma [170]. These results have laid the groundwork for expansion of TCR into other cancer types. In breast cancer, an HLA-A*O2-restricted TCR targeted p53^R175H^ therapy from peripheral blood lymphocytes was infused into a patient, who experienced a partial response lasting 6 months [171]. Upcoming trials of TCR in breast cancer include a phase Ib trial (NCT05989828) for patients with metastatic TNBC which expresses HLA-A*O2 restricted NY-ESO-1, a phase I trial (NCT05877599) of HLA-A*02 restricted NT-175 in solid tumors including breast cancer, and two phase I studies (NCT05483491, NCT05035407) of HLA-A*01-restricted KK-LC-1 in solid tumors including breast cancer (Table 8).

**Table 8:** Current and upcoming clinical trials of cellular therapy in breast cancer

Trial name	Primary author	Year	Study design	Line of therapy	Setting	TAAs/MOA	# Patients	Drug regimen	Results
*TIL therapy*									
NCT01174121	Zacharakis	2022	Phase I/II	Heavily pretreated	Metastatic		6	TILs + pembrolizumab + IL-2	Response in 3/6 patients, with one complete response and 2 partial responses
*CAR-T cell therapy*									
NCT01837602	Tchou	2017	Phase 0	Previously treated	Metastatic		6	Intratumoral c-MET CAR-T cells	No clinical responses seen six grade-3 AE; no CRS
NCT03060356	Shah	2023	Phase I	Heavily pretreated	Metastatic TNBC		4	Intravenous C-MET CAR-T cells	2/4 patients with stable disease No Grade 3 or higher AE
NCT04727151	Schlecter	2023	Phase I	Heavily pretreated	Advanced HER2 + Solid Malignancy	T cell Antigen Coupler	18	TAC01-HER2	Tolerable safety
*TCR Therapy*									
NCT03412877	Kim	2022	Phase II	Heavily pretreated	Advanced Breast Cancer	HLA-A*02/p53R175H	1	HLA-A*O2-restricted TCR targeted p53R175H	Partial reponse lasting 6 mo
*Selected Upcoming Clinical Trials*									
NCT04111510			Phase II	Second line	mTNBC	TIL LN-145	6	TIL LN-145	ORR
NCT05451784			Phase I/II	Maximum 5 prior lines of therapy	a/m TNBC	TIL	20	PD-L1+ TILS	ORR and Safety
NCT03449108			Phase II	Previously treated	solid tumors including TNBC	TIL LN-145-S1	30	Ipilumumab + Nivolumab + cyclophosphamide + fludarabine + IL-2 + LN-145-S1	ORR
NCT05576077			Phase Ib	Previously treated	solid tumors including MBC	TIL TBio-4101	60	TBio-4101 + Pembrolizumab	Safety
NCT02830724			Phase I/II	Previously treated	solid tumors expressing CD70	CD70 CAR-T cells	124	CD70 CAR-T cells	Safety + ORR
NCT06010862			Phase I	Third line	solid tumors expressing CEA	CEA CAR-T cells	36	CEA CAR-T cells	Safety, Adverse Events
NCT06126406			Phase I	Third line	solid tumors expressing CEA	CEA CAR-T cells	60	CEA CAR-T cells	Safety, Adverse Events
NCT06043466			Phase I	Heavily pretreated	solid tumors expressing CEA	CEA CAR-T cells	30	CEA CAR-T cells	DLT
NCT04348643			Phase I/II	Previously treated	solid tumors expressing CEA	CEA CAR-T cells	40	CEA CAR-T cells	Safety
NCT04107142			Phase I	Previously treated	solid tumors, including TNBC	CTM-N2D CAR-T cells	10	CTM-N2D CAR-T cells	Safety
NCT02915445			Phase I	Third line	solid tumors expressing EpCAM	EpCAM CAR-T cells	30	EpCAM CAR-T cells	DLT
NCT03635632 GAIL-N			Phase I	Previously Treated	solid tumors expressing GD2	GD2 CAR-T Cells	94	GD2 CAR-T Cells	Safety
NCT03696030			Phase I	Previously treated	solid tumors expressing HER2 with brain and/or leptomeningeal metastases	HER2 CAR-T	39	intraventricular HER2-CAR T cells	DLT
NCT02442297			Phase I	Previously treated	solid tumors expressing HER2 with brain metastasis	HER2 CAR-T cells	10	Intracranial HER2 CAR-T cells	Safety
NCT03740256 VISTA			Phase I	Heavily pretreated	solid tumors expressing HER2	HER2 CAR-T cells + oncolytic adenovirus	45	HER2 CAR-T cells + intratumoral CAdVEC	Safety
NCT06251544 TRAILBLASER			Phase I	> First Line	Metastatic Breast Cancer (HER2 1+ , 2+ , or 3+)	HER2 and TR2 targeting CAR T cells + IL-15	27	Arm A: HTR2 T Cells (without lymphodepletion) Arm B: HTR2 T Cells (with lymphodepletion)	DLT
NCT02414269			Phase I/II	> First line	mesothelioma, breast or lung cancer with pleural disease	CAR-T cells expressing mesothelin	113	Arm A: intrapleural iCasp9M28z CAR-T cells Arm A: intrapleural iCasp9M28z CAR-T cells + chemotherapy Arm A: intrapleural iCasp9M28z CAR-T cells + pembrolizumab	AE and CBR
NCT02792114			Phase I	> First line	HER2- MBC expressing mesothelin	Mesothelin-targeting CAR-T cells	186	Mesothelin-targeting CAR-T cells	MTD
NCT02792114			Phase I	Previously treated	HER2- MBC expressing mesothelin	Mesothelin CAR-T cells	186	Mesothelin CAR-T	Safety
NCT02580747			Phase I	Previously treated	solid tumors expressing mesothelin, including TNBC	Mesothelin CAR-T cells	20	Mesothelin CAR-T cells	Safety
NCT05623488			Phase I	Previously treated	TNBC expressing mesothelin	Mesothelin CAR-T cells	12	huCART-meso CAR-T cells	Safety
NCT02587689			Phase I/II	Heavily pretreated	solid tumors expressing MUC1, including TNBC	MUC1 CAR-T cells	20	MUC1 CAR-T cells	Safety
NCT04020575			Phase I/II	Heavily pretreated	metastatic MUC1* + breast cancer	MUC-1 CAR-T	69	CAR T-targeting MUC1*	Safety
NCT05239143			Phase I	Heavily pretreated	solid tumors expressing MUC1-c	MUC1 CAR-T cells	100	Arm A: P-MUC1C-ALLO1 CAR-T cells in single ascending dose with chemo 1 Arm A: P-MUC1C-ALLO1 CAR-T cells in cyclical ascending dose with chemo 1 Arm A: P-MUC1C-ALLO1 CAR-T cells in single ascending dose with chemo 2 Arm A: P-MUC1C-ALLO1 CAR-T cells in cyclical ascending dose with chemo 2	Safety and Tolerability Prelininary Efficacy
NCT04430595			Phase I/II	Previously treated	a/m breast cancer expressing GD2, CD44v6, or Her2	Multispecific CAR-T cells	100	Multispecific CAR‐T cells targeting HER2, CD2 and CD44v6	Safety
NCT02706392			Phase I	Heavily pretreated	Metastatic TNBC expressing ROR1	ROR1 CAR-T cells	21	ROR1 CAR-T cells	Safety
NCT04119024			Phase I	Heavily pretreated	Metastatic Melanoma or solid tumors	IL13Ralpha2/CD19	18	IL13Ralpha2 CAR T Cells	Safety, DLT
NCT05274451			Phase I	Previously treated	solid tumors expressing ROR1 including TNBC	ROR1 CAR-T cells	100	LYL797 CAR-T cells	DLT
NCT04427449			Phase I/II	Previously treated	advanced malignancy expressing CD44v6	4SCAR-CD44v6 CAR-T cells	100	4SCAR-CD44v6 CAR-T cells	Safety + ORR
NCT05877599			Phase I	Heavily pretreated	TP53 R175H mutant solid tumors including MBC	TCR	162	anti-HLA-A2/NT-175	Safety
NCT05989828			Phase Ib	Previously treated	Metastatic TNBC expressing NY-ESO-1	TCR	20	cyclophosphamide + fludarabine + IL-2 + anti-HLA-A2/NY-ESO-1 TCR-T cells	MTD
NCT01967823			Phase II	Previously treated	Metastatic solid tumors expressing NY-ESO-1, including breast cancer	TCR	11	cyclophosphamide + fludarabine + IL-2 + anti-HLA-A2/NY-ESO1 TCR-T cells	ORR
NCT05296564			Phase I/II	Previously treated	Metastatic cancer expressing NY-ESO-1, including TNBC	TCR	43	cyclophosphamide + fludarabine + IL-2 + anti-HLA-A2/NY-ESO1 TCR-T cells	Safety, Response Rate
NCT03159585			Phase I	Previously treated	Metastatic cancer expressing NY-ESO-1, including breast cancer	TCR	6	cyclophosphamide + fludarabine + anti-HLA-A2/NY-ESO1 TCR-T cells	Safety
NCT05035407			Phase I	Previously Treated	solid tumors including MBC	TCR	100	cyclophosphamide + fludarabine + IL-2 + anti-HLA-A01/KK-LC-1 TCR	Safety
NCT06253520			Phase I	Previously treated	solid tumors including breast cancer	TCR	210	cyclophosphamide + fludarabine + IL-2 + HLA restrcted/KRAS TCR-T cells + vaccine	Safety, Clinical Response Rate

## Future outlook and conclusions

The use of immunotherapy for the treatment of breast cancer has expanded over the last two decades. Pembrolizumab is a premier example of an immune checkpoint inhibitor that has changed treatment paradigms for TNBC, while ongoing clinical trials investigate the role of pembrolizumab and other immune checkpoint inhibitors in patients with HR+ and HER2+ disease. Antibody–drug conjugates such as sacitizumab govitecan and trastuzumab deruxtecan have ushered in a new generation of anti-cancer therapies, with ongoing studies evaluating new antibody–drug conjugates such as datopotamab deruxtecan as well as antibody–drug conjugates in combination with immune checkpoint inhibitors. With fourteen bispecifics approved for solid tumors to date, further studies of bispecifics such as zanidatamab and zenocutuzmab in breast cancer are needed to clarify the role for these agents in the current treatment paradigm. Future studies should investigate mechanisms to enhance the T cell response, whether through dual checkpoint inhibition and/or costimulatory signals, in both bispecifics and CAR-T therapy. Upcoming clinical trials bring hope for broadening the treatment landscape and further understanding the potential benefit of combination immune checkpoint inhibitors, antibody–drug conjugates, bispecific antibodies, and/or cellular therapies. Further research regarding biomarkers beyond PD-L1 expression and other characteristics of the tumor immune microenvironment will enhance our ability to predict which patients will respond to immune checkpoint inhibitor-containing treatments, allowing for more tailored therapy for each individual patient.

## Data Availability

No datasets were generated or analysed during the current study.

## Abbreviations

TIME: Tumor immune microenvironment
PD-1: Programmed-death 1
PD-L1: Programmed-death ligand 1
CTLA4: Cytotoxic T-lymphocyte associate protein 4
ICIs: Immune checkpoint inhibitors
LAG-3: Lymphocyte activation gene 3
ADC: Antibody drug conjugate
TAA: Tumor associated antigen
CPS: Combined positive score
IC: Immune-cell score
PD-L1+: Programmed-death ligand 1 positive
TILs: Tumor infiltrating lymphocytes
TMB: Tumor mutational burden
dMMR: Deficiencies in mismatch repair genes
MSI-H: Microsatellite instability-high
irAE: Immune-related adverse events
TNBC: Triple negative breast cancer
HR+: Hormone receptor positive
a/m: Advanced/metastatic
ORR: Overall response rate
PFS: Progression free survival
OS: Overall survival
HR: Hazard ratio
DFI: Disease-free interval
IIT: Intention-to-treat
PARPi: Poly (ADP-ribose) polymerase inhibitors
mBC: Metastatic breast cancer
SG: Sacituzumab govitecan
Dato-DXd: Datopotamab deruxtecan
pCR: Pathologic complete response
DFS: Disease-free survival
RCB: Residual cancer burden
EFS: Event-free survival
Dd: Dose-dense
AC: Doxorubicin and cyclophosphamide
PC: Paclitaxel and carboplatin
EC: Epirubicin and cyclophosphamide
KLH: Keyhole limpet hemocyanin
T-VEC: Talimogene iaherparepvec
LV: Ladoratizimab vedotin
CDK4/6i: CDK4/6 inhibitor
ILD: Interstitial lung disease
T-AC: Paclitaxel followed by doxorubicin and cyclophosphamide
IHC: Immunohistochemical
T-DXd: Trastuzumab deruxtecan
T-Duo: Trastuzumab duocarmazine
CAR-T: Chimeric antigen receptor
TCR: T cell receptor engineered
HLA: Human leukocyte antigen
MHC: Major histocompatibility complex
Afami-cel: Afamitresgene autoleucel
Lete-cel: Letetresgene autoleucel
